# Association between dietary B vitamin intakes and advanced cardiovascular-kidney-metabolic syndrome: insights from the National Health and Nutrition Examination Survey and an external hospital-based dataset

**DOI:** 10.3389/fnut.2026.1901987

**Published:** 2026-08-17

**Authors:** Dingyuan Tu, Jinru Li, Li Luo, Jianming Wang

**Affiliations:** 1 Department of Congenital Heart Disease, General Hospital of Northern Theater Command, Shenyang, Liaoning, China; 2 Department of Cardiology, The 961st Hospital of the Joint Logistics Support Force of The Chinese People’s Liberation Army, Qiqihar, Heilongjiang, China; 3 Department of Anesthesiology, The 961st Hospital of the Joint Logistics Support Force of The Chinese People’s Liberation Army, Qiqihar, Heilongjiang, China

**Keywords:** B vitamin intakes, cardiovascular-kidney-metabolic syndrome, National Health and Nutrition Examination Survey, quantile-based g-computation, weighted quantile sum

## Abstract

**Background:**

Advanced cardiovascular-kidney-metabolic (CKM) syndrome is highly prevalent. Diet is increasingly considered as a modifiable risk factor for advanced CKM syndrome. This study aims to evaluate the associations of B vitamin intakes with advanced CKM syndrome.

**Methods:**

A cross-sectional study was performed on participants in 2011 to 2020 National Health and Nutrition Examination Survey. Participants were categorized into five CKM stages (0–4) according to the different clinical severities of CKM syndrome, with stages 3 and 4 considered advanced. B vitamin intakes were assessed through two 24-h dietary recall interviews. Multivariable weighted logistic regression, restricted cubic spline analysis, weighted quantile sum (WQS) regression and quantile-based g-computation (QG-comp) were used to explore the associations of single B vitamin intake and mixed B vitamin intakes with advanced CKM syndrome. The primary findings were then externally validated in an independent Chinese cohort.

**Results:**

A total of 14,576 CKM syndrome patients were included in the final analysis, of which 2,486 were categorized into advanced CKM syndrome. Multivariable weighted logistic regression showed that higher intakes of vitamin B2 (odds ratio [OR], 0.87; 95% confidence interval [CI], 0.81–0.93) and B6 (OR, 0.88; 95% CI, 0.82–0.95) were associated with a lower likelihood of advanced stages in CKM syndrome patients. Meanwhile, WQS regression and QG-comp analyses consistently indicated a negative association between mixed B vitamin intakes and advanced CKM syndrome, highlighting that vitamin B2 and vitamin B6 were the most significant factors driving the overall effect. These associations were consistently replicated in the external validation cohort.

**Conclusion:**

Higher intakes of mixed B vitamins were associated with lower risk of advanced CKM syndrome, with vitamin B2 and vitamin B6 emerging as the primary contributors.

## Introduction

Cardiovascular disease (CVD) ranks as the primary cause of death worldwide and is a major contributor to health problems worldwide (1). As heart disease and stroke statistics of 2025 report from American Heart Association (AHA), CVD affects 48.6% of U.S. adults aged 20 and above, and its prevalence grows with age among both men and women (2). There is a rising awareness of the scientific basis for the complex relationships among CVD, chronic kidney disease (CKD), and metabolic diseases (3, 4). The AHA introduced the cardiovascular-kidney-metabolic (CKM) syndrome construct in an October 2023 presidential advisory, offering a unified framework that captures the pathophysiological interplay among metabolic diseases, CKD, and CVD (5). This syndrome is stratified into five stages (0 to 4) based on accumulating risk factors and established disease burden. According to recent epidemiological estimates using NHANES data, approximately 90% of U.S. adults meet criteria for at least stage 1 CKM syndrome, while 15% have progressed to advanced stages (stage 3 or 4) (6). Furthermore, a higher CKM stage has been prospectively associated with elevated all-cause mortality risk (7). The poor CKM health creates a heavy economic and social burden. Hence, identifying factors associated with advanced CKM stages is critical for generating hypotheses to guide future prevention and intervention strategies.

Dietary risk plays a critical role in the development of CVD, CKD and metabolic disease. In 2021, poor dietary habits were responsible for 31.3% of the burden of CVD, 17.9% of CKD, and 24.2% of diabetes mellitus (8). Among the dietary nutrients, B vitamins are crucial micronutrients necessary for various metabolic functions important for human health. They act as co-factors for a variety of enzymes involved in energy metabolism, protein synthesis, and other key functional pathways (9). Imbalanced levels of B vitamins result in intracellular oxidative stress and endothelial dysfunction, which subsequently initiate a range of chronic diseases, including CVD (10), CKD (11), and diabetes mellitus (12). However, the association between B vitamin intakes and CKM syndrome progression is still unclear.

To clarify the association of different B vitamin intakes with advanced CKM syndrome, we investigated the independent and combined cross-sectional associations of multiple B vitamin intakes with the presence of advanced CKM syndrome in the 2011–2020 National Health and Nutrition Examination Survey (NHANES). We then externally validated the primary findings in an independent Chinese cohort to assess the robustness and generalizability of the associations.

## Methods

### Study design and participant selection

The NHANES is a biennial, cross-sectional program administered by the National Center for Health Statistics. It employs a stratified, multistage probability cluster sampling design to generate nationally representative estimates for the civilian, non-institutionalized U.S. population. NHANES consists of two parts which are in-home personal interviews followed by a standardized physical examination carried out in mobile examination centers. The research protocol for NHANES was approved by the National Center for Health Statistics and Ethics Review Board. All participants provided written informed consent. The detailed methodology and protocols are available on the NHANES website^1^.

**Figure 1:**
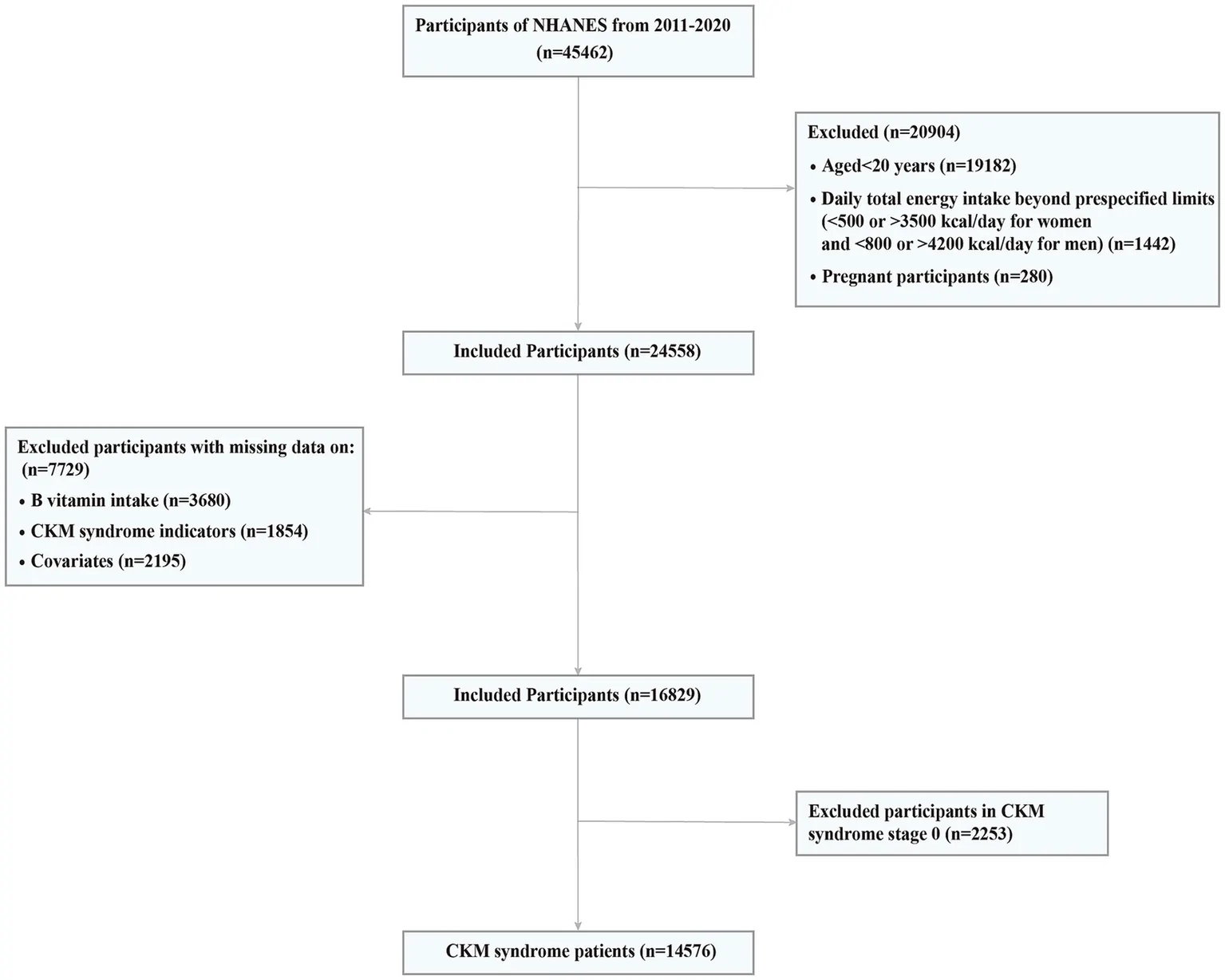
Flow diagram of the screening and enrollment of study participants in NHANES. CKM, cardiovascular-kidney-metabolic; NHANES, National Health and Nutrition Examination Survey.

Since NHANES began categorizing Asian race separately starting in the 2011–2012 cycle, we enrolled adult participants of NHANES (2011–2020) aged 20 years and above who completed at least one valid dietary recall. Individuals were not included in the study if they fulfilled any of the following criteria: (1) pregnant at baseline; (2) those who had total energy intake beyond prespecified limits (600–3,600 kcal/day for women and 800–4,200 kcal/day for men) (13); (3) missing B vitamin intake data; (4) missing information on CKM syndrome indicators; (5) participants with missing covariates. In the end, a total of 16,829 participants were enrolled (Figure 1).

For external validation, we recruited an independent cohort from the 961st Hospital of the Joint Logistics Support Force of The Chinese People’s Liberation Army between January 1, 2025 and December 31, 2025. The study protocol was approved by the hospital’s ethics committee (Approval No. 2025–016), and written informed consent was obtained from all participants. The inclusion and exclusion criteria were identical to those applied in the NHANES cohort. A total of 368 individuals were enrolled. The detailed participant recruitment and selection process is presented in Supplementary Figure S1.

### Measurement of B vitamin intake

The U.S. Department of Agriculture created and confirmed a multiple-pass dietary recall method for NHANES to collect dietary information. Participants reported all food and beverages consumed in two 24-h dietary recall periods. The first dietary recall was conducted in-person in mobile examination centers, and the second dietary recall was conducted 3–10 days later via telephone. Based on the quantity of food and beverages reported and the corresponding nutrient contents by the National Center for Health Statistics, the caloric content and other nutrients derived from each consumed food and beverage item were calculated (14). Our analysis focused on six specific B vitamins: vitamin B1, vitamin B2, vitamin B6, vitamin B12, niacin, and total folate. The average intake for each B vitamin was calculated across the 2 days.

In the external validation cohort, dietary intake was assessed during face-to-face interviews using a validated semi-quantitative food frequency questionnaire (FFQ). The accuracy of this FFQ for estimating energy and nutrient intakes has been previously confirmed (15, 16). Participants were thoroughly questioned about all foods consumed, whether eaten at home or away. Nutrient intakes were calculated using the China Food Composition Table (17). We also focused on the same six B vitamins: vitamin B1, vitamin B2, vitamin B6, vitamin B12, niacin, and total folate.

### Definitions of the components of CKM syndrome

According to the AHA criteria, CKM syndrome stages are determined by 10 components: body mass index (BMI), waist circumference, hypertension, diabetes, prediabetes, hypertriglyceridemia, metabolic syndrome, CKD risk stage, 10-year CVD risk, and clinical CVD (5). BMI was calculated as weight in kilograms divided by height in meters squared. Hypertension was indicated by a systolic blood pressure of 140 mmHg or more, a diastolic blood pressure of 90 mmHg or more, a self-reported history of high blood pressure, or the consumption of antihypertensive drugs. Diabetes was identified by a fasting blood glucose ≥125 mg/dL, a Hemoglobin A1c ≥ 6.5%, self-reported history of diabetes, or current hypoglycemic treatment. Prediabetes was defined as having a fasting blood glucose level from 100 to 124 mg/dL, or a Hemoglobin A1c of 5.7 to 6.4%. Metabolic syndrome is characterized by having any three of these five criteria: (1) the fasting blood glucose >100 mg/dL or drug treatment for diabetes mellitus; (2) high-density lipoprotein (HDL) cholesterol <50 mg/dL in females, <40 mg/dL in males or drug treatment for reduced HDL cholesterol; (3) plasma triglyceride >150 mg/dL or drug treatment for raised triglyceride; (4) waist circumference >88 cm in women or >102 cm in men; (5) blood pressure >130/85 mmHg or drug treatment for raised blood pressure. According to the Kidney Disease Improving Global Outcome criteria, CKD status was classified into low risk, moderate risk, high risk, and very high risk (Supplementary Table S1) based on estimated glomerular filtration rate (eGFR) and urinary albumin-creatinine ratio (UACR) (5). It should be noted that the NHANES data rely on a single measurement of eGFR and UACR, which cannot confirm the chronicity of kidney disease. This has the potential to misclassify individuals with transient renal dysfunction or albuminuria as having CKD risk, which may lead to misclassification of CKM stages. Besides, 10-year CVD risk was estimated with the Framingham risk score (18).

### Definitions of CKM syndrome stages

The CKM syndrome staging followed the AHA Presidential Advisory criteria (5), with adaptations for NHANES data availability as previously described by Aggarwal et al. (6). Briefly, stage 0 indicates no CKM risk factors (normal BMI, waist circumference, blood pressure, glucose, lipids, and no CKD or CVD). Stage 1 involves excess adiposity or prediabetes. Stage 2 encompasses metabolic risk factors (hypertriglyceridemia, hypertension, metabolic syndrome, or diabetes) or moderate-to-high-risk CKD. Stage 3 is defined by subclinical CVD (10-year CVD risk ≥20% using the Framingham risk score) or very-high-risk CKD. Stage 4 denotes established clinical CVD. The detailed descriptions of CKM syndrome stage definitions are in Supplementary Table S2. Consistent with prior reports, we defined CKM syndrome as stage 1 or higher, and advanced CKM syndrome as stages 3 or 4 (6).

### Covariate assessments

For Covariates, we extracted demographic and lifestyle covariates from household interview data, including age, sex, ethnicity (non-Hispanic White, non-Hispanic Black, non-Hispanic Asian, Hispanic, or other), educational attainment (less than high school, high school graduate, or college degree or higher), family income-to-poverty ratio (<1, 1–3, >3), marital status (coupled [married/living with partner] vs. single/separated), smoking status (never, former, current), alcohol status (non-drinker, current low-to-moderate drinker, current heavy drinker), physical activity level, and overall diet quality. Definitions for smoking and drinking categories followed standard NHANES criteria detailed elsewhere (19). Physical activity level was defined according to the frequency and intensity of participant’s self-reported leisure-time physical activity in the past month, and classified into sufficient and insufficient, based on metabolic equivalent intensity levels (20). The Healthy Eating Index-2015 (HEI-2015), which scores from 0 to 100 based on a participant’s 24-h dietary recall, was utilized to assess overall diet quality, where higher scores reflect better diet quality (21). In the external validation cohort, covariates included age, sex, and marital status.

### Statistical analysis

The analyses incorporated complex survey design aspects like sample weights, clustering, and stratification, adhering to NHANES data instructions (22). Baseline comparisons stratified by advanced CKM syndrome were performed using a weighted t-test for continuous variables and chi-square test for categorical variables. Continuous variables were presented as weighted mean value with weighted standard errors (SE), and categorical variables as absolute numbers with associated weighted percentages. Three survey-weighted logistic regression models were applied to estimate odds ratios (OR) and corresponding 95% confidence interval (CI) for the association between six B vitamins and advanced CKM syndrome. Model 1 was adjusted for age, sex, and ethnicity; Model 2 added adjustments for educational level, family income, and marital status; Model 3 further included adjustments for smoking status, alcohol status, physical activity, and HEI-2015. Moreover, we used restricted cubic spline (RCS) model to investigate the potential dose–response relationships (linear or non-linear) between continuous B vitamins and advanced CKM syndrome. To achieve a balance between optimal fitting and overfitting of the principal spline, the number of knots was chosen to be between three and seven, guided by the minimum absolute value of the Akaike information criterion. In addition, to further explore the relationship between vitamin B and advanced CKM syndrome, the study divided vitamin B levels into quartiles.

In order to address the problem of multicollinearity and to distinguish between the health effects of each B vitamin, we used two novel and complementary approaches, weighted quantile sum (WQS) regression and quantile-based g-computation (QG-comp) models, to comprehensively investigate the overall effects of six B vitamins on the risk of advanced CKM syndrome. The WQS regression summarizes the joint effect of multiple chemicals by creating a weighted index of correlated chemical exposures that are weighted according to their strength of association with the outcome (23). To estimate the WQS index and the adaptive weights, the dataset was randomly split into a training dataset (40%) and a validation dataset (60%). In this study, we used 100 iterations of the repeated holdout WQS regression model proposed by Tanner (24) with 500 bootstraps per WQS regression iteration. The relative contribution of each B vitamin to the overall effect was quantified by the estimated weights, where larger weights indicate greater influence on the joint association with the outcome. Nevertheless, WQS regression is confined to measuring outcome-related exposures in a single direction. To address this constraint, we introduced the QG-comp model, which estimates positive and negative weights separately for each mixture component without assuming a homogeneous direction of effect, allowing us to identify the strongest contributors in each direction (25).

For the external validation cohort, analyses did not incorporate sampling weights, as the cohort was recruited via convenience sampling and did not follow a complex survey design. Baseline characteristics were compared using Student’s *t*-test or Mann–Whitney *U* test for continuous variables and Chi-square test for categorical variables, as appropriate. Logistic regression models were fitted to explore the association between six B vitamins and advanced CKM syndrome.

Finally, several sensitivity analyses were performed to improve the reliability of our study. First, participants who had self-reported a cancer history were excluded. Second, excluding eligible participants with missing covariate data could cause selection bias. To tackle this issue, we did iterative imputation for missing covariate data using the machine learning algorithm in the missRanger package in R. Third, considering the potential association of medication with CKM syndrome, we excluded participants who were treated with sodium-glucose cotransporter-2 inhibitors and glucagon-like peptide 1 receptor agonists (5). Fourth, given that CKM syndrome stages are ordinal categories (stage 0 through 4), we performed ordinal logistic regression as an alternative analytical approach to examine the association between B vitamin intakes and CKM syndrome stages. This analysis preserved the ordinal nature of the outcome variable and served as a sensitivity analysis to complement our primary binary logistic regression models. Besides, to examine whether the associations between B vitamin intakes and advanced CKM syndrome were modified by age and sex, we performed stratified analyses by age groups (20–44, 45–64, and ≥65 years) and sex. Multiplicative interaction terms (age × B vitamin and sex × B vitamin) were added to the fully adjusted models to formally test for effect modification. A *p*-value for interaction < 0.05 was considered statistically significant.

All analyses were conducted using R version 4.4.2, including the “survey,” “gWQS,” “qgcomp,” and “rms” packages. A *p*-value of less than 0.05 was deemed statistically significant in a two-tailed test.

## Results

### Baseline characteristics of patients according to advanced CKM syndrome

Of the 16,829 adults from NHANES 2011 to 2020, CKM syndrome stage 0 to 4 accounted for 13.83, 28.13, 46.09, 4.13 and 11.14%, respectively. We then removed 2,253 participants in CKM stage 0, and finally this study included 14,576 CKM syndrome patients (Figure 1). Table 1 shows the baseline characteristics of CKM syndrome patients, with a mean (SE) age of 49.89 (0.31) years and 51.29% female. Compared to non-advanced CKM syndrome patients, advanced CKM syndrome patients were more likely to be older, male, non-Hispanic white, former and current smokers, and non-drinkers, have lower level of education, less high-income households, lower physical activity levels, as well as higher overall diet quality. Additionally, advanced CKM syndrome patients exhibited lower daily B vitamins intake levels for vitamin B2, vitamin B6, and niacin, while no significant differences were observed for the other B vitamins (vitamin B1, vitamin B12, and total folate).

**Table 1:** Baseline characteristics of patients with cardiovascular-kidney-metabolic syndrome.

Characteristic	Overall (*N* = 14,576)	Non-advanced CKM (*N* = 12,090)	Advanced CKM (*N* = 2,486)	*p-*value
Age (years), mean (SE)	49.89(0.31)	46.71(0.28)	66.28(0.34)	<0.0001
Sex, *n* (%)				<0.0001
Female	7,462(51.29)	6,395(52.65)	1,067(44.24)	
Male	7,114(48.71)	5,695(47.35)	1,419(55.76)	
Race or ethnicity, *n* (%)				<0.0001
Hispanic	3,417(13.97)	3,065(15.53)	352(5.92)	
Non-Hispanic White	5,776(67.88)	4,275(65.14)	1,501(81.99)	
Non-Hispanic Black	3,371(10.53)	2,903(11.10)	468(7.59)	
Non-Hispanic Asian	1,481(4.39)	1,386(4.94)	95(1.57)	
Other	531(3.22)	461(3.28)	70(2.94)	
Education level, *n* (%)				<0.0001
Less than high school	2,912(13.21)	2,303(12.52)	609(16.77)	
High school or equivalent	3,283(22.70)	2,636(21.87)	647(26.97)	
College or above	8,381(64.09)	7,151(65.61)	1,230(56.26)	
Family PIR, *n* (%)				<0.0001
<1.0	2,987(13.54)	2,453(13.38)	534(14.36)	
1.0–3.0	6,079(36.25)	4,891(34.83)	1,188(43.60)	
>3.0	5,510(50.21)	4,746(51.79)	764(42.04)	
Marital, *n* (%)				0.46
Coupled	8,729(64.89)	7,333(65.28)	1,396(62.85)	
Single or separated	5,847(35.11)	4,757(34.72)	1,090(37.15)	
Smoking status, *n* (%)				<0.0001
Never	8,162(55.32)	7,146(58.22)	1,016(40.35)	
Former	3,696(26.90)	2,736(24.59)	960(38.83)	
Now	2,718(17.78)	2,208(17.19)	510(20.81)	
Alcohol status, *n* (%)				<0.0001
Non-drinker	3,947(22.55)	3,013(20.30)	934(34.19)	
Low to moderate drinker	7,861(57.24)	6,569(57.69)	1,292(54.92)	
Heavy drinker	2,768(20.21)	2,508(22.01)	260(10.89)	
Physical activity, *n* (%)				<0.0001
Sufficient	3,207(24.74)	2,985(27.56)	222(10.17)	
Insufficient	11,369(75.26)	9,105(72.44)	2,264(89.83)	
HEI-2015, mean (SE)	50.71(0.24)	50.46(0.25)	52.00(0.42)	<0.001
Vitamin B1 (mg), mean (SE)	1.55(0.01)	1.55(0.01)	1.52(0.02)	0.18
Vitamin B2 (mg), mean (SE)	2.09(0.02)	2.10(0.02)	2.03(0.03)	0.03
Vitamin B6 (mg), mean (SE)	2.07(0.02)	2.10(0.02)	1.90(0.04)	<0.001
Vitamin B12 (mg), mean (SE)	4.86(0.09)	4.91(0.10)	4.59(0.14)	0.08
Niacin (mg), mean (SE)	25.19(0.19)	25.64(0.21)	22.89(0.36)	<0.0001
Total folate (μg), mean (SE)	383.85(3.13)	386.37(3.39)	370.85(7.54)	0.06

Values are weighted mean (weighted standard errors) for continuous variables or numbers (weighted %) for categorical variables.

CKM, Cardiovascular-Kidney-Metabolic; HEI-2015, Healthy Eating Index-2015; PIR, poverty income ratio.

To examine the potential impact of excluding participants with missing dietary data, we compared the baseline characteristics between the 3,680 excluded participants and the 16,829 included participants in the NHANES cohort (Supplementary Table S3). Compared with the included participants, those excluded were older, had lower socioeconomic status, and importantly, had a higher proportion of advanced CKM syndrome stages (stage 3: 14.57% vs. 4.87%; stage 4: 13.31% vs. 8.79%). This suggests that participants with more severe CKM syndrome were more likely to have missing dietary recall data, which may have led to an underrepresentation of advanced cases in our analytic sample.

In the external validation cohort, after excluding participants with CKM syndrome stage 0, a total of 323 CKM syndrome patients (stage ≥1) were included in the analysis. The baseline characteristics stratified by advanced CKM syndrome are summarized in Supplementary Table S4. Consistent with the NHANES findings, patients with advanced CKM syndrome were older, more likely to be male, and had significantly lower daily intakes of vitamin B2, vitamin B6, and niacin compared to non-advanced patients. No significant differences were observed for vitamin B1, vitamin B12, or total folate. These patterns mirrored those seen in the NHANES cohort, supporting the reproducibility of the associations.

### Association of single B vitamin intake with advanced CKM syndrome

Table 2 summarizes the continuous analyses between six B vitamin intakes and advanced CKM syndrome. In the full multivariable-adjusted model (Model 3), after adjustment for age, sex, ethnicity, educational level, family income, marital status, smoking status, alcohol status, physical activity, and HEI-2015, vitamin B2, vitamin B6, and niacin were negatively associated with advanced CKM syndrome, and the OR (95% CI) were 0.87 (0.81, 0.93), 0.88 (0.82, 0.95) and 0.98 (0.97, 0.99), respectively. Vitamin B1 (OR: 0.92, 95% CI: 0.84–1.01, *p* = 0.09), vitamin B12 (OR: 0.99, 95% CI: 0.97–1.00, *p* = 0.12), and total folate (OR: 1.00, 95% CI: 1.00–1.00, *p* = 0.21) showed no significant association with advanced CKM syndrome.

**Table 2:** Association between continuous B vitamin intakes and advanced CKM syndrome.

Vitamin B	Model 1		Model 2		Model 3	
	OR (95% CI)	*p*-value	OR (95% CI)	*p*-value	OR (95% CI)	*p*-value
Vitamin B1						
Per 1 unit increment	0.89(0.82,0.97)	<0.01	0.92(0.84,0.99)	<0.05	0.92(0.84,1.01)	0.09
Vitamin B2						
Per 1 unit increment	0.86(0.80,0.92)	<0.0001	0.88(0.83,0.94)	<0.001	0.87(0.81,0.93)	<0.001
Vitamin B6						
Per 1 unit increment	0.90(0.85,0.97)	0.004	0.86(0.80,0.93)	<0.001	0.88(0.82,0.95)	0.001
Vitamin B12						
Per 1 unit increment	0.98(0.96,1.00)	0.11	0.99(0.97,1.01)	0.15	0.99(0.97,1.00)	0.12
Niacin						
Per 1 unit increment	0.99(0.98,0.99)	0.003	0.99(0.98,0.99)	0.02	0.98(0.97,0.99)	0.04
Total folate						
Per 1 unit increment	1.00(1.00,1.00)	0.02	1.00(1.00,1.00)	0.09	1.00(1.00,1.00)	0.21

Values are weighted OR (95% CI).

Model 1: adjusted for age, sex, and ethnicity.

Model 2: Model 1 + educational level, family income, and marital status.

Model 3: Model 2 + smoking status, alcohol status, physical activity, and HEI-2015.

CI, Confidence interval; CKM, Cardiovascular-Kidney-Metabolic; HEI-2015, Healthy Eating Index-2015; OR, Odds ratios.

Additionally, the levels of the six B vitamins were divided into quartiles, and the results are illustrated in Table 3. Compared to the lowest quartile (Q1), the OR of advanced CKM syndrome was decreased in the highest quantile (Q4) by 27% of vitamin B2 (OR: 0.73, 95%CI: 0.60–0.89), 26% of vitamin B6 (OR: 0.74, 95%CI: 0.59–0.93), and 18% of vitamin B12 (OR: 0.82, 95%CI: 0.70–0.97), respectively.

**Table 3:** Association between quartiles of B vitamin intakes and advanced CKM syndrome.

Vitamin B	Model 1		Model 2		Model 3	
	OR (95% CI)	*p*-value	OR (95% CI)	*p*-value	OR (95% CI)	*p*-value
Vitamin B1						
Q1	1(reference)		1(reference)		1(reference)	
Q2	1.00(0.83,1.19)	0.96	1.05(0.88,1.25)	0.59	1.10(0.92,1.31)	0.30
Q3	0.71(0.59,0.87)	0.001	0.76(0.62,0.92)	0.01	0.78(0.64,0.95)	0.02
Q4	0.84(0.70,1.01)	0.07	0.91(0.75,1.10)	0.31	0.93(0.77,1.13)	0.48
*P* for the trend	0.01		0.05		0.09	
Vitamin B2						
Q1	1(reference)		1(reference)		1(reference)	
Q2	0.93(0.78,1.12)	0.46	1.00(0.83,1.21)	0.96	1.01(0.84,1.21)	0.92
Q3	0.68(0.58,0.79)	<0.0001	0.77(0.65,0.92)	0.004	0.78(0.65,0.92)	0.004
Q4	0.67(0.56,0.80)	<0.0001	0.75(0.63,0.90)	0.002	0.73(0.60,0.89)	0.002
*P* for the trend	<0.0001		<0.0001		<0.0001	
Vitamin B6						
Q1	1(reference)		1(reference)		1(reference)	
Q2	0.83(0.70,0.99)	0.04	0.88(0.74,1.05)	0.15	0.92(0.78,1.10)	0.37
Q3	0.68(0.56,0.82)	<0.001	0.76(0.63,0.92)	0.01	0.81(0.66,0.99)	0.04
Q4	0.62(0.51,0.77)	<0.0001	0.69(0.56,0.86)	<0.001	0.74(0.59,0.93)	0.01
*P* for the trend	<0.0001		<0.001		0.01	
Vitamin B12						
Q1	1(reference)		1(reference)		1(reference)	
Q2	0.75(0.61,0.93)	0.01	0.79(0.65,0.98)	0.03	0.82(0.67,1.01)	0.06
Q3	0.90(0.76,1.05)	0.18	0.95(0.80,1.12)	0.54	0.97(0.82,1.16)	0.76
Q4	0.74(0.63,0.87)	<0.001	0.80(0.68,0.94)	0.01	0.82(0.70,0.97)	0.02
*P* for the trend	0.01		0.06		0.11	
Niacin						
Q1	1(reference)		1(reference)		1(reference)	
Q2	0.92(0.81,1.05)	0.24	0.99(0.87,1.13)	0.87	1.01(0.88,1.16)	0.88
Q3	0.75(0.64,0.89)	<0.001	0.81(0.69,0.96)	0.02	0.84(0.71,0.99)	0.03
Q4	0.74(0.59,0.94)	0.01	0.83(0.65,1.05)	0.12	0.88(0.67,1.10)	0.22
*P* for the trend	0.03		0.04		0.09	
Total folate						
Q1	1(reference)		1(reference)		1(reference)	
Q2	0.81(0.66,0.99)	0.04	0.85(0.69,1.04)	0.11	0.89(0.72,1.09)	0.25
Q3	0.71(0.59,0.85)	<0.001	0.77(0.64,0.92)	0.01	0.81(0.67,0.98)	0.03
Q4	0.66(0.55,0.79)	<0.0001	0.73(0.60,0.89)	0.002	0.78(0.64,0.96)	0.02
*P* for the trend	<0.0001		<0.001		0.01	

Values are weighted OR (95% CI).

Model 1: adjusted for age, sex, and ethnicity.

Model 2: Model 1 + educational level, family income, and marital status.

Model 3: Model 2 + smoking status, alcohol status, physical activity, and HEI-2015.

Abbreviations: CI, Confidence interval; CKM, Cardiovascular-Kidney-Metabolic; HEI-2015, Healthy Eating Index-2015; OR, Odds ratios.

**Figure 2:**
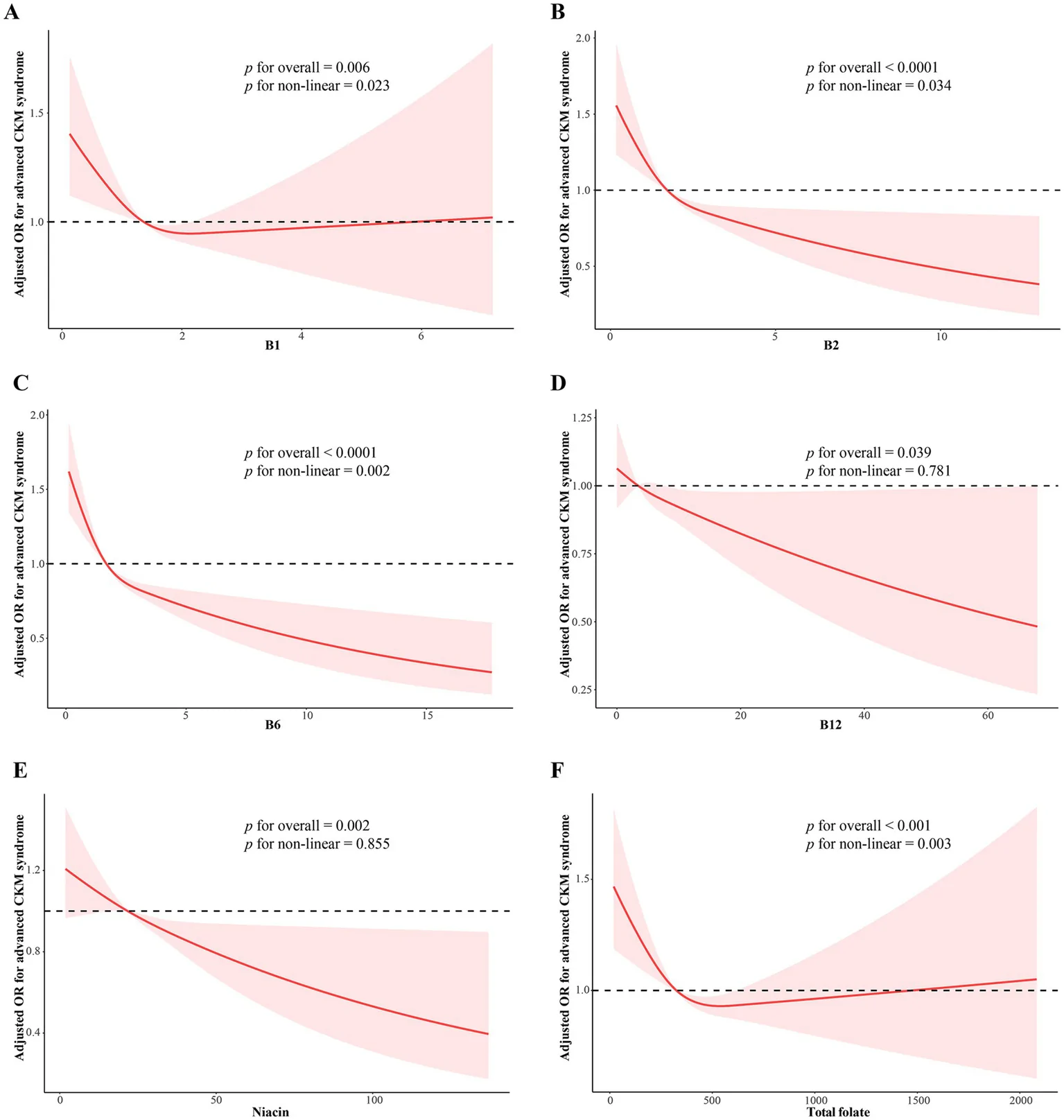
The weighted restricted cubic spline plots for single B vitamin intake and advanced CKM syndrome. **(A)** The association of vitamin B1 and advanced CKM syndrome. **(B)** The association of vitamin B2 and advanced CKM syndrome. **(C)** The association of vitamin B6 and advanced CKM syndrome. **(D)** The association of vitamin B12 and advanced CKM syndrome. **(E)** The association of niacin and advanced CKM syndrome. **(F)** The association of total folate and advanced CKM syndrome. The models were adjusted for all covariates. Abbreviation: CKM, cardiovascular-kidney-metabolic.

We further estimated the dose–response relationship between six B vitamin intakes and advanced CKM syndrome with RCS functions (Figure 2). A nonlinear correlation between vitamin B1, vitamin B2, vitamin B6, total folate and advanced CKM syndrome was confirmed using RCS regression (*p*-value for non-linearity: *p* = 0.023 for vitamin B1, *p*-value = 0.034 for vitamin B2, *p*-value = 0.002 for vitamin B6, *p* = 0.003 for total folate). Conversely, the results from RCS analysis showed that vitamin B12 (*p*-value for nonlinearity = 0.781) and niacin (*p*-value for non-linearity = 0.855) did not show a non-linear correlation with advanced CKM syndrome, but instead exhibited a significant linear correlation.

We then examined the same associations in the external validation cohort. As shown in Supplementary Table S5, each 1-unit increment in vitamin B2 intake was associated with a 14% reduction in the odds of advanced CKM syndrome (OR 0.86, 95% CI 0.80–0.92, *p* < 0.0001), vitamin B6 with a 10% reduction (OR 0.90, 95% CI 0.85–0.97, *p* = 0.004), and niacin with a 1% reduction (OR 0.99, 95% CI 0.98–0.99, *p* = 0.003). Vitamin B1, Vitamin B12 and total folate were not consistently associated with advanced CKM syndrome, though the *p*-value for total folate was nominally significant due to very narrow confidence intervals.

Quartile analyses in the validation cohort (Supplementary Table S5) further supported the inverse association between higher B vitamin intakes and advanced CKM syndrome. Compared with the lowest quartile (Q1), participants in the highest quartile (Q4) had significantly lower odds of advanced CKM syndrome for vitamin B2 (OR 0.76, 95% CI 0.68–0.97), vitamin B6 (OR 0.78, 95% CI 0.68–0.96), and total folate (OR 0.82, 95% CI 0.69–0.98). No significant trends were detected for vitamin B1, vitamin B12, or niacin. These quartile-based results were largely consistent with the continuous analyses, reinforcing the robustness of the inverse associations for vitamin B2 and B6. We further performed age- and sex-stratified analyses to examine whether the associations varied across demographic subgroups. As shown in Supplementary Tables S6–S11, the inverse associations between vitamin B2 and B6 intakes and advanced CKM syndrome were generally consistent across age groups and between sexes. Importantly, none of the interaction terms reached statistical significance (all *p* for interaction > 0.05 for both age and sex across all six B vitamins), indicating that the observed associations did not differ significantly by age or sex.

Overall, the external validation cohort confirmed the key findings from NHANES. Higher intakes of vitamin B2 and B6 were consistently associated with a lower likelihood of advanced CKM syndrome.

### Associations of mixed B vitamin intakes with advanced CKM syndrome

**Figure 3:**
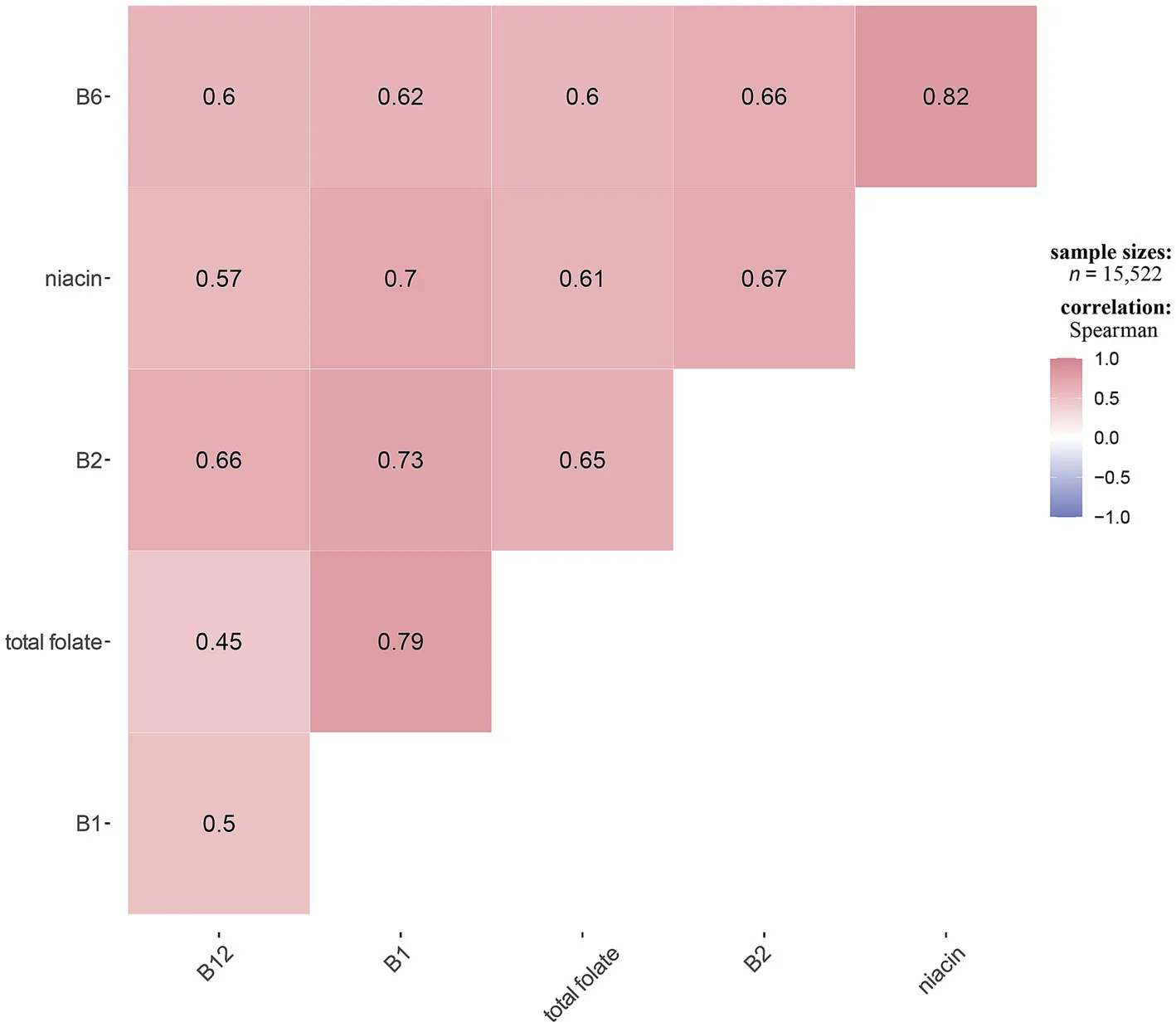
The Spearman correlation between six B vitamin intakes.

In our study, we utilized the Spearman correlation coefficient to analyze the interaction among various vitamin B types (Figure 3). The relationship between the six B vitamins was statistically significant (correlation coefficient r ranging from 0.45 to 0.82; *p* < 0.05). The findings indicated the highest correlation was between vitamin B6 and niacin with a correlation coefficient of 0.82. Strong correlations were also observed between vitamin B1 and folate (*r* = 0.79), as well as between vitamin B1 and vitamin B2 (*r* = 0.73). However, there was a low correlation of 0.45 between vitamin B12 and folate.

**Figure 4:**
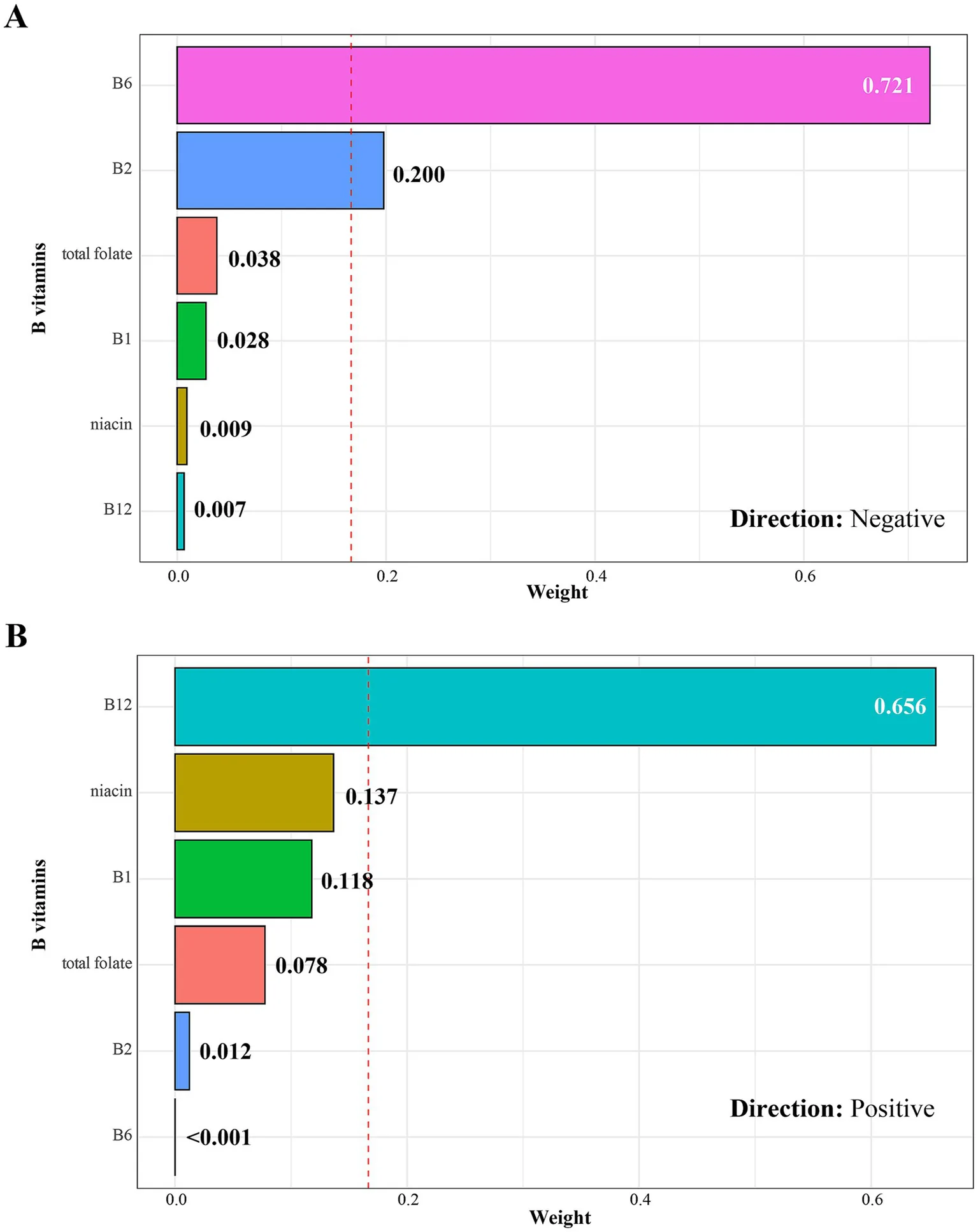
Estimated weights of six B vitamin intakes for advanced CKM syndrome by WQS models. **(A)** The WQS model weights of B vitamin intakes on advanced CKM syndrome in negative direction. **(B)** The WQS model weights of B vitamin intakes on advanced CKM syndrome in positive direction. The models were adjusted for all covariates. Abbreviation: CKM, cardiovascular-kidney-metabolic; WQS, weighted quantile sum.

To further investigate the combined impact of mixed B vitamin intakes on the risk of advanced CKM syndrome, we developed a WQS regression model. The mixed effects of six B vitamins co-exposure showed a significant negative correlation with advanced CKM syndrome risk (OR: 0.89, 95% CI: 0.83–0.95, *p* < 0.001), indicating a significant inverse association between B vitamins co-exposure and the odds of advanced CKM syndrome. The estimated B vitamin weight of each WQS index was presented in Figure 4. In the B vitamin mixtures, vitamin B6 (weighted index: 0.721) and vitamin B2 (weighted index: 0.200) were found to be the most significant contributors, indicating that these two vitamins carried the greatest weight in driving the overall inverse association. Meanwhile, the positive WQS model revealed that vitamin B12 (weighted index: 0.656) contributed the highest weight in increasing the risk of advanced CKM syndrome.

**Figure 5:**
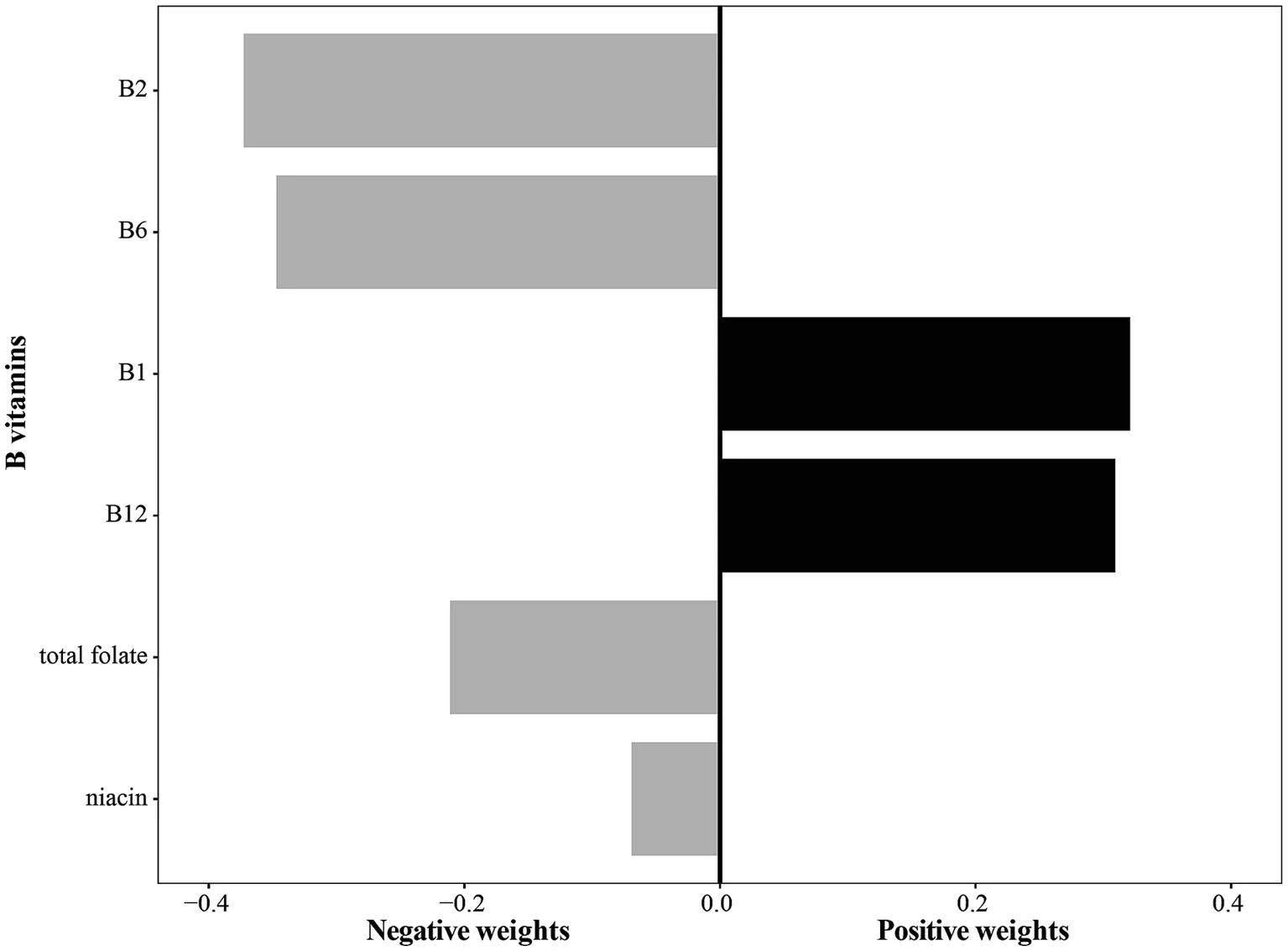
Quantile g-computation regression analysis of the relationship between six B vitamin intakes and advanced CKM syndrome. The model was adjusted for all covariates.

Similar to the findings described above, the result of QG-comp model revealed that the joint intakes of mixed B vitamins were significantly associated with a decreased risk of advanced CKM syndrome (OR: 0.89, 95% CI: 0.83–0.94, *p* < 0.0001). The estimated weights of the positive and negative factors were displayed in Figure 5, where vitamin B2 (weighted index: 0.373) was the strongest negative driver, followed by vitamin B6 (weighted index: 0.347). The consistency of these weighting patterns across the two complementary models strengthens our confidence that these vitamins were the primary contributors. In addition, vitamin B1 had the greatest positive weighted index of 0.321, followed by vitamin B12 (weighted index: 0.309).

### Sensitivity analyses

In the sensitivity analyses, the relationship between mixed B vitamin intakes and advanced CKM syndrome remained robust after excluding participants with baseline self-reported history of cancer, iterative imputation on covariates, and excluding participants treated with sodium-glucose cotransporter-2 inhibitors and glucagon-like peptide 1 receptor agonists. In addition, ordinal logistic regression analyses treating CKM syndrome stages (0 through 4) as an ordinal outcome yielded results consistent with our primary binary logistic regression models, further confirming the robustness of the findings (Supplementary Tables S12–S20).

## Discussion

Analyzing data from 14,576 U.S. adults with CKM syndrome, this cross-sectional investigation employed survey-weighted logistic regression, RCS, WQS regression, and QG-comp models to assess both individual and combined associations of six dietary B vitamins with the likelihood of being at advanced CKM stages. Compared to non-advanced CKM syndrome patients, advanced CKM syndrome patients had significantly lower daily intake levels of vitamin B2, vitamin B6, and niacin. Single logistic regression models indicated that higher intakes of vitamin B2 and B6 were inversely associated with advanced stages in CKM syndrome patients, as well as significant trends among quartiles. Moreover, both the two mixed models (WQS and QG-comp) consistently demonstrated a negative relationship between mixed B vitamin intakes and advanced CKM syndrome, while vitamin B2 and vitamin B6 were the most significant factors driving the overall effect. These findings were further validated in an independent external cohort, where the inverse associations for vitamin B2 and B6 were consistently replicated. These cross-sectional findings indicated a significant inverse association between B vitamin intakes and the presence of advanced CKM syndrome. While causality cannot be inferred, these results identify B vitamins, particularly B2 and B6, as candidate nutrients that warrant further investigation in prospective studies as potential factors for maintaining CKM health.

B vitamins are an essential group of micronutrients, acting as crucial cofactors for many enzymes or directly involved in host metabolism. They influence a broad range of physiological functions in the human body, spanning from energy production to the regulation of the immune system (26). Diet is a key factor that influence the circulating levels of B vitamins. Overall, inadequate B vitamin intakes have been associated with chronic diseases. For example, higher vitamin B1 intake was associated with a trend toward lower risk of hypertension, heart failure and CVD mortality (27). A shortage intake of vitamin B2, a precursor of flavin mononucleotide and flavin adenine dinucleotide, may lead to multi-system disorders such as diabetes (28, 29) and gastric cancer (30). Deficiency in vitamin B6 intake has been linked to a broad spectrum of diseases, including CVD (31, 32), diabetes (33), frailty (34), and the risk of all-cause and CVD mortality (35). Besides, dietary supplementation with vitamin B12 and folate could have therapeutic potential for the prevention or treatment of non-alcoholic steatohepatitis (36). The connections between B vitamin intakes and chronic diseases should be given enough attention in the context of the increasing global burden of obesity, diabetes, metabolic syndrome, CKD, and CKD, all of which are key components of CKM syndrome. Since a healthy diet has been identified as a fundamental step in the prevention of CKM syndrome progression (5), our study provided the first population-based evidence, albeit cross-sectional, of an association between higher B vitamin intakes and a lower likelihood of being in advanced CKM stages.

According to the AHA Presidential Advisory Statement (5) and the epidemiological study of CKM syndrome (6), advanced CKM syndrome stages were defined as stages 3 or 4 because the two stages identify individuals with subclinical or clinical CVD. One of the main goals of CKM syndrome management is to identify optimal strategies for halting or reversing its progression (5). Recent studies have identified some blood indexes to be related to advanced CKM syndrome. A national cohort study of 6,858 Chinese middle-aged and older adults found that individuals with higher remnant cholesterol had a significantly increased risk of advanced CKM stages (37). Another study in the NHANES 2011–2018 revealed that elevated triglyceride glucose index increased the odds of advanced stages in CKM syndrome patients (38). Additionally, some lifestyle factors are connected with advanced CKM syndrome. Our previous research displayed that compared to CKM syndrome patients in the low sleep quality group, those with a high and moderate sleep quality had a 45 and 32% lower risk of being in advanced stages, respectively (39). Based on the China Cardiovascular Disease and Cancer Cohort, Li et al. (40) discovered that those adhering to optimal Life’s Essential 8 health behavior had lower risks of advanced CKM syndrome. Our study extends these findings by showing that B vitamin intake is inversely associated advanced CKM syndrome, highlighting the significance of considering B vitamin intake as a modifiable lifestyle factor for prevention of advanced CKM syndrome stage.

Since B vitamins are commonly present in a variety of foods and can potentially interact (41), we applied the WQS index to indicate the collective influence of mixed B vitamin intakes. The WQS model aimed to evaluate the impact of each variable on the overall effect by creating weighted quartiles for a single exposure. It outperforms traditional regression methods by successfully addressing collinearity and variance inflation issues. Furthermore, to counter the limitation of the directional homogeneity assumption in the WQS regression model, the QG-comp model was utilized afterward as a supplementary tool. Taken these two complementary approaches as a whole, we discovered that among the six specific B vitamins, vitamin B2 and vitamin B6 exhibited the strongest inverse associations with advanced CKM syndrome. The potential mechanisms linking vitamin B2 and B6 to CKM syndrome progression may be understood through several interconnected pathways that are central to cardiovascular-renal-metabolic pathology. First, both vitamins play critical roles in one-carbon metabolism, which provides methyl groups for DNA and histone methylation (42). These epigenetic modifications can influence gene expression pathways involved in insulin sensitivity, lipid metabolism, and inflammatory responses. Disruption of one-carbon metabolism due to inadequate B vitamin intake may therefore contribute to the development of insulin resistance and metabolic dysregulation, key drivers of CKM syndrome (5). Second, vitamin B2 and B6 possess potent antioxidant properties that are particularly relevant to the shared pathophysiology of CKM syndrome. Vitamin B2, as a precursor of flavin mononucleotide and flavin adenine dinucleotide, is essential for the glutathione redox cycle, a major cellular antioxidant defense system that protects against lipid peroxidation and oxidative injury (43). Vitamin B6 has been shown to mitigate oxidative stress by restoring ornithine aminotransferase expression, thereby reducing the production of reactive oxygen species (44). Given that oxidative stress is a common pathogenic mechanism underlying endothelial dysfunction, vascular stiffness, and kidney injury, the antioxidant actions of these vitamins may help protect against the progressive deterioration across multiple organ systems in CKM syndrome (45, 46). Third, the interplay between oxidative stress and inflammation is particularly relevant to the cardio-renal-metabolic axis. Reactive oxygen species promote the activation of pro-inflammatory pathways, which in turn exacerbate insulin resistance, promote atherosclerotic plaque formation, and accelerate kidney fibrosis (47). By reducing oxidative burden, vitamin B2 and B6 may attenuate this vicious cycle, thereby slowing the progression from early CKM risk factors to advanced stages (48, 49). However, given the cross-sectional nature of our study, these mechanistic interpretations remain speculative and require confirmation through experimental and longitudinal studies.

This study has a number of strengths. First, this study utilized a complex multistage probability sampling method to ensure participants were representative of the general population, allowing the results to be generalized across the U.S. Second, our study employed various statistical models, significantly enhancing the reliability of the conclusions. Third, and most importantly, the key findings were independently validated in an external cohort with a different cultural and dietary background. This two-cohort design, with discovery followed by replication, substantially strengthens the robustness and generalizability of the observed associations beyond a single dataset. However, there are several potential limitations that need to be acknowledged. First, a critical consideration in interpreting our findings is the potential for reverse causation. Due to the cross-sectional design, we cannot determine whether lower B vitamin intake preceded or resulted from the development of advanced CKM syndrome. The observed associations could reflect either direction of the relationship, namely low intake promoting CKM progression or CKM progression leading to reduced intake due to illness-related dietary changes. Patients with established CVD, CKD, or diabetes may have altered their dietary habits following diagnosis, potentially under medical advice to restrict certain foods. Furthermore, severe illness and poor appetite associated with these conditions can lead to reduced total energy and nutrient intake. Consequently, the observed inverse association between B-vitamin intakes and advanced CKM might reflect, at least in part, the impact of disease on dietary behavior rather than a protective effect of these micronutrients. Only a longitudinal study with repeated dietary assessments can disentangle the direction of this association. Second, the assessment of B vitamin intakes was based on dietary recall interviews in the NHANES cohort and a food frequency questionnaire in the validation cohort, both of which are susceptible to recall bias and may not fully capture habitual long-term intake. In addition, our analysis quantified B vitamin intake exclusively from dietary sources and did not include the contribution from supplements. While the NHANES cohort collected information on supplement use, the external validation cohort did not have comparable data. To maintain consistency in exposure measurement across the two cohorts, we focused on dietary B vitamin intake as the common metric available in both datasets. This approach may have led to an underestimation of total B vitamin intake and potentially diluted the strength of the observed associations. Future studies incorporating both dietary and supplemental sources are warranted. Third, in the NHANES cohort, we excluded participants with missing dietary recall data (n = 3,680) to ensure data completeness. As shown in Supplementary Table S3, these excluded participants had a higher proportion of advanced CKM stages compared with the included participants, suggesting that the most severe cases were more likely to be excluded due to missing dietary data. This exclusion would likely bias our effect estimates toward the null (i.e., underestimate the true association), because the excluded group with a higher burden of advanced disease would have contributed to a stronger inverse association if they had been included. However, this exclusion criterion was not applied to the external validation cohort, in which all participants had complete FFQ data, and the consistent findings across both cohorts help mitigate concerns about selection bias. Nevertheless, future studies with more complete dietary data collection are warranted to further confirm our results. Fourth, the definition of CKD risk was based on a single point measurement of eGFR and UACR, which may not reflect chronic conditions and could lead to the misclassification of individuals with acute kidney injury or transient proteinuria into higher CKM stages. This misclassification would likely bias our effect estimates toward the null or in an unpredictable direction. Fifth, certain CVD-related data, like echocardiographic parameters and coronary angiography, which are used to define advanced CKM syndrome stages, were not available in the NHANES database, potentially causing an underestimation of advanced CKM syndrome. Sixth, although we adjusted for a comprehensive set of covariates including overall diet quality (HEI-2015), residual confounding from unmeasured factors such as supplement use, health literacy, or access to care cannot be ruled out. Seventh, in the external validation cohort, the adjustment for covariates was limited to age, sex, and marital status due to the constraints of data collection in the hospital setting, whereas the NHANES cohort adjusted for a broader set of confounders including socioeconomic status, lifestyle factors, and overall dietary quality. This discrepancy may introduce residual confounding in the validation analyses. Nonetheless, the fact that the inverse associations for vitamin B2 and B6 remained significant in the validation cohort despite this limited adjustment suggests that the findings are unlikely to be entirely attributable to unmeasured confounders. Future external validation studies with more comprehensive covariate collection are warranted to further confirm these results. Finally, although the discovery cohort was based on a single U.S. population and the external validation cohort was recruited from a hospital setting rather than a community-based sample, we externally validated the main findings in an independent Chinese cohort. The consistent replication of the inverse associations between vitamin B2 and B6 intakes and advanced CKM syndrome across these two distinct populations, which differ substantially in sampling framework, geographic region, ethnicity, and dietary patterns, supports the generalizability of our findings. Nonetheless, further validation in other populations and settings is still warranted.

## Conclusion

In conclusion, in this nationally representative sample of U.S. adults, our study revealed that higher intakes of several B vitamins, notably vitamin B2 and vitamin B6, were independently associated with a lower likelihood of advanced CKM syndrome. Mixed models consistently demonstrated a significant negative association between joint B vitamin intakes and advanced CKM syndrome, with vitamin B2 and vitamin B6 as the primary contributors. Findings were consistently replicated in an independent Chinese validation cohort. Given the inherent limitations of cross-sectional design in establishing temporality, prospective cohort studies are required to determine the predictive value of B vitamin intakes for the incidence and progression of CKM syndrome. Moreover, further experimental research is urgently needed to examine the underlying molecular mechanisms.

## Data Availability

The raw data supporting the conclusions of this article will be made available by the authors, without undue reservation.

## Footnotes

1
https://www.cdc.gov/nchs/nhanes/index.html

## References

[1] Chong B Jayabaskaran J Jauhari SM Chan SP Goh R Kueh MTW . Global burden of cardiovascular diseases: projections from 2025 to 2050. Eur J Prev Cardiol. (2024) 32:1001–15. doi: 10.1093/eurjpc/zwae281, 39270739

[2] Martin SS Aday AW Allen NB Almarzooq ZI Anderson CAM Arora P . 2025 heart disease and stroke statistics: a report of US and global data from the American Heart Association. Circulation. (2025) 151:e41–e660. doi: 10.1161/cir.0000000000001303, 39866113 PMC12256702

[3] Ndumele CE Neeland IJ Tuttle KR Chow SL Mathew RO Khan SS . A synopsis of the evidence for the science and clinical management of cardiovascular-kidney-metabolic (CKM) syndrome: a scientific statement from the American Heart Association. Circulation. (2023) 148:1636–64. doi: 10.1161/CIR.0000000000001186 37807920

[4] Sebastian SA Padda I Johal G. Cardiovascular-kidney-metabolic (CKM) syndrome: a state-of-the-art review. Curr Probl Cardiol. (2024) 49:102344. doi: 10.1016/j.cpcardiol.2023.102344, 38103820

[5] Ndumele CE Rangaswami J Chow SL Neeland IJ Tuttle KR Khan SS . Cardiovascular-kidney-metabolic health: a presidential advisory from the American Heart Association. Circulation. (2023) 148:1606–35. doi: 10.1161/CIR.0000000000001184 37807924

[6] Aggarwal R Ostrominski JW Vaduganathan M. Prevalence of cardiovascular-kidney-metabolic syndrome stages in US adults, 2011-2020. JAMA. (2024) 331:1858–60. doi: 10.1001/jama.2024.6892, 38717747 PMC11079779

[7] Li N Li Y Cui L Shu R Song H Wang J . Association between different stages of cardiovascular-kidney-metabolic syndrome and the risk of all-cause mortality. Atherosclerosis. (2024) 397:118585. doi: 10.1016/j.atherosclerosis.2024.118585, 39255681

[8] G.R.F. Collaborators. Global burden and strength of evidence for 88 risk factors in 204 countries and 811 subnational locations, 1990-2021: a systematic analysis for the global burden of disease study 2021. Lancet. (2024) 403:2162–203. doi: 10.1016/S0140-6736(24)00933-4 38762324 PMC11120204

[9] Piquereau J Boitard SE Ventura-Clapier R Mericskay M. Metabolic therapy of heart failure: is there a future for B vitamins? Int J Mol Sci. (2021) 23:30. doi: 10.3390/ijms23010030, 35008448 PMC8744601

[10] Yuan S Mason AM Carter P Burgess S Larsson SC. Homocysteine, B vitamins, and cardiovascular disease: a mendelian randomization study. BMC Med. (2021) 19:97. doi: 10.1186/s12916-021-01977-8, 33888102 PMC8063383

[11] Xu X Qin X Li Y Sun D Wang J Liang M . Efficacy of folic acid therapy on the progression of chronic kidney disease. JAMA Intern Med. (2016) 176:1443–50. doi: 10.1001/jamainternmed.2016.4687, 27548766

[12] Zhu Y Ying T Xu M Chen Q Wu M Liu Y . Joint B vitamin intake and type 2 diabetes risk: the mediating role of inflammation in a prospective Shanghai cohort. Nutrients. (2024) 16:1901. doi: 10.3390/nu16121901, 38931256 PMC11206684

[13] Cao Y Willett WC Rimm EB Stampfer MJ Giovannucci EL. Light to moderate intake of alcohol, drinking patterns, and risk of cancer: results from two prospective US cohort studies. BMJ. (2015) 351:h4238. doi: 10.1136/bmj.h4238, 26286216 PMC4540790

[14] Bleich SN Wang YC Wang Y Gortmaker SL. Increasing consumption of sugar-sweetened beverages among US adults: 1988–1994 to 1999–2004. Am J Clin Nutr. (2009) 89:372–81. doi: 10.3945/ajcn.2008.26883, 19056548

[15] Lan QY Zhang YJ Liao GC Zhou RF Zhou ZG Chen YM . The association between dietary vitamin a and carotenes and the risk of primary liver Cancer: a case-control study. Nutrients. (2016) 8:624. doi: 10.3390/nu8100624, 27727160 PMC5084012

[16] Zhu J Bo Y Ma R Jiang Z Wang J Yuan Z . Association between dietary choline intake and odds of preeclampsia: a case-control study. Front Nutr. (2025) 12:1703117. doi: 10.3389/fnut.2025.1703117, 41262735 PMC12623378

[17] Yang WG Pan X. China Food Composition, vol. 42. Beijing, China: Peking University Medical Press (2009). p. 795–9.

[18] D'Agostino RB Sr Vasan RS Pencina MJ Wolf PA Cobain M Massaro JM . General cardiovascular risk profile for use in primary care: the Framingham heart study. Circulation. (2008) 117:743–53. doi: 10.1161/CIRCULATIONAHA.107.699579, 18212285

[19] Tu D Ju S Xue Y Xie W Wu C Ma C . Association between asthma and advanced cardiovascular-kidney-metabolic syndrome in U.S. adults, a cross-sectional study from NHANES 2011–2023. Respir Med. (2025) 247:108288. doi: 10.1016/j.rmed.2025.108288, 40752629

[20] Beddhu S Baird BC Zitterkoph J Neilson J Greene T. Physical activity and mortality in chronic kidney disease (NHANES III). Clin J Am Soc Nephrol. (2009) 4:1901–6. doi: 10.2215/CJN.01970309, 19820134 PMC2798872

[21] Krebs-Smith SM Pannucci TE Subar AF Kirkpatrick SI Lerman JL Tooze JA . Update of the healthy eating index: HEI-2015. J Acad Nutr Diet. (2018) 118:1591–602. doi: 10.1016/j.jand.2018.05.021, 30146071 PMC6719291

[22] N.C.f.H. Statistics, NHANES Survey Methods and Analytic Guidelines. Hyattsville, Maryland. (2013).

[23] Carrico C Gennings C Wheeler DC Factor-Litvak P. Characterization of weighted quantile sum regression for highly correlated data in a risk analysis setting. J Agric Biol Environ Stat. (2014) 20:100–20. doi: 10.1007/s13253-014-0180-3, 30505142 PMC6261506

[24] Tanner EM Hallerbäck MU Wikström S Lindh C Kiviranta H Gennings C . Early prenatal exposure to suspected endocrine disruptor mixtures is associated with lower IQ at age seven. Environ Int. (2020) 134:105185. doi: 10.1016/j.envint.2019.105185, 31668669

[25] Keil AP Buckley JP O’Brien KM Ferguson KK Zhao S White AJ. A quantile-based g-computation approach to addressing the effects of exposure mixtures. Environ Health Perspect. (2020) 128:47004. doi: 10.1289/EHP5838, 32255670 PMC7228100

[26] Yang Y Ke Y Liu X Zhang Z Zhang R Tian F . Navigating the B vitamins: dietary diversity, microbial synthesis, and human health. Cell Host Microbe. (2024) 32:12–8. doi: 10.1016/j.chom.2023.12.004, 38211561

[27] Wen H Niu X Zhao R Wang Q Sun N Ma L . Association of vitamin B1 with cardiovascular diseases, all-cause and cardiovascular mortality in US adults. Front Nutr. (2023) 10:1175961. doi: 10.3389/fnut.2023.1175961, 37720374 PMC10502219

[28] Yang H Wang X Xi X Xia Y Jiang M Zuo H. Effect modification of the association between vitamin B2 intake and diabetes mellitus by sex: findings from the National Health and nutrition examination survey 2013–2020. Front Nutr. (2024) 11:1510096. doi: 10.3389/fnut.2024.1510096, 39737150 PMC11682993

[29] Sawicki CM Haslam DE Braun KVE Drouin-Chartier J-P Voortman T Franco OH . Methyl donor nutrient intake and incidence of type 2 diabetes: results from three large U.S. cohorts. Diabetes Care. (2023) 46:1799–806. doi: 10.2337/dc23-0662, 37643330 PMC10516245

[30] Lu YT Gunathilake M Lee J Choi IJ Kim Y-I Kim J. Riboflavin intake, MTRR genetic polymorphism (rs1532268) and gastric cancer risk in a Korean population: a case–control study. Br J Nutr. (2021) 127:1026–33. doi: 10.1017/s0007114521001811 34078503

[31] Jayedi A Zargar MS. Intake of vitamin B6, folate, and vitamin B12 and risk of coronary heart disease: a systematic review and dose-response meta-analysis of prospective cohort studies. Crit Rev Food Sci Nutr. (2018) 59:2697–707. doi: 10.1080/10408398.2018.1511967, 30431328

[32] Wang C Li B Zhu Q Zhang Q Xie Z Xie H . Dietary vitamin B6 intake and stroke are negatively associated in adults: a cross-sectional study from the NHANES. Heliyon. (2024) 10:e31125. doi: 10.1016/j.heliyon.2024.e31125, 38778939 PMC11109891

[33] Horikawa C Aida R Kamada C Fujihara K Tanaka S Tanaka S . Vitamin B6 intake and incidence of diabetic retinopathy in Japanese patients with type 2 diabetes: analysis of data from the Japan diabetes complications study (JDCS). Eur J Nutr. (2019) 59:1585–94. doi: 10.1007/s00394-019-02014-4, 31152214

[34] Cheng X Hu Y Ruan Z Zang G Chen X Qiu Z. Association between B-vitamins intake and frailty among patients with chronic obstructive pulmonary disease. Aging Clin Exp Res. (2023) 35:793–801. doi: 10.1007/s40520-023-02353-7, 36719551

[35] Zhao L-G Shu X-O Li H-L Gao J Han L-H Wang J . Prospective cohort studies of dietary vitamin B6 intake and risk of cause-specific mortality. Clin Nutr. (2019) 38:1180–7. doi: 10.1016/j.clnu.2018.04.016, 29764693 PMC6551204

[36] Tripathi M Singh BK Zhou J Tikno K Widjaja A Sandireddy R . Vitamin B12 and folate decrease inflammation and fibrosis in NASH by preventing syntaxin 17 homocysteinylation. J Hepatol. (2022) 77:1246–55. doi: 10.1016/j.jhep.2022.06.033, 35820507

[37] Ding X Tian J Chang X Liu J Wang G. Association between remnant cholesterol and the risk of cardiovascular-kidney-metabolic syndrome progression: insights from the China health and retirement longitudinal study. Eur J Prev Cardiol. (2025) 32:1157–1165. doi: 10.1093/eurjpc/zwaf248 40272444

[38] Wu L Huang Z. Elevated triglyceride glucose index is associated with advanced cardiovascular kidney metabolic syndrome. Sci Rep. (2024) 14:31352. doi: 10.1038/s41598-024-82881-y, 39732891 PMC11682451

[39] Tu D Sun J Wang P Xu Q Ma C. Overall sleep quality is associated with advanced stages in patients with cardiovascular–kidney–metabolic syndrome. J Am Heart Assoc. (2025) 14:e038674. doi: 10.1161/JAHA.124.038674, 40130386 PMC12132850

[40] Li M Xu M Ding Y Lin H Qin G Wang T . Life’s essential 8 cardiovascular health, cardiovascular-kidney-metabolic syndrome stages, and incident cardiovascular events: a nationwide 10-year prospective cohort study in China. Cardiovasc Diabetol. (2025) 24:197. doi: 10.1186/s12933-025-02735-3, 40346555 PMC12065188

[41] Paul L Selhub J. Interaction between excess folate and low vitamin B12 status. Mol Asp Med. (2017) 53:43–7. doi: 10.1016/j.mam.2016.11.004, 27876554

[42] Keating ST Plutzky J El-Osta A. Epigenetic changes in diabetes and cardiovascular risk. Circ Res. (2016) 118:1706–22. doi: 10.1161/CIRCRESAHA.116.306819, 27230637 PMC5593074

[43] Ashoori M Saedisomeolia A. Riboflavin (vitamin B2) and oxidative stress: a review. Br J Nutr. (2014) 111:1985–91. doi: 10.1017/S0007114514000178, 24650639

[44] Shen H Zhou L Yang Y Shu H Wu D Yang S . Gut microbiota-produced vitamin B6 mitigates alcohol-associated liver disease by attenuating hepatic oxidative stress damage. Hepatol Commun. (2025) 9:e0599. doi: 10.1097/HC9.0000000000000599, 39670862 PMC11637752

[45] Ning R Li Y Du Z Li T Sun Q Lin L . The mitochondria-targeted antioxidant MitoQ attenuated PM2.5-induced vascular fibrosis via regulating mitophagy. Redox Biol. (2021) 46:102113. doi: 10.1016/j.redox.2021.102113, 34425389 PMC8379696

[46] Adeshara K Di Marco E Bordino M Gordin D Bernardi L Cooper ME . Altered oxidant and antioxidant levels are associated with vascular stiffness and diabetic kidney disease in type 1 diabetes after exposure to acute and chronic hyperglycemia. Cardiovasc Diabetol. (2024) 23:350. doi: 10.1186/s12933-024-02427-4, 39342285 PMC11439198

[47] Jankauskas SS Komici K Varzideh F Aversa LS Kansakar U D'Onghia ML . Cardiovascular-kidney-metabolic syndrome: a comprehensive review of pathophysiology, epidemiology, diagnosis, and management. Cardiovasc Diabetol. (2026) 25:182. doi: 10.1186/s12933-026-03177-1 42057054 PMC13274062

[48] Mazur-Bialy AI Pocheć E. Vitamin B2 deficiency enhances the pro-inflammatory activity of adipocyte, consequences for insulin resistance and metabolic syndrome development. Life Sci. (2017) 178:9–16. doi: 10.1016/j.lfs.2017.04.010, 28414075

[49] Munteanu C Schwartz B. B vitamins, glucoronolactone and the immune system: bioavailability, doses and efficiency. Nutrients. (2023) 16:24. doi: 10.3390/nu16010024, 38201854 PMC10780850

