# Supplementary material for: Association between dietary B vitamin intakes and advanced cardiovascular-kidney-metabolic syndrome: insights from the National Health and Nutrition Examination Survey and an external hospital-based dataset
Source: Front Nutr. 2026 Aug 17;13:1901987. doi: 10.3389/fnut.2026.1901987 (PMC13526985; doi:10.3389/fnut.2026.1901987)
Supplement: Supplementary file 1 [file Data_sheet_1.docx]

**Figure S1** Flow diagram of the screening and enrollment of study participants in the external validation cohort.

Abbreviation: CKM, cardiovascular-kidney-metabolic.

**Table S1** KDIGO risk for CKD classification.

| **eGFR, ml/min per 1.73 m2** | **Urinary ACR, mg/g** | | |
| --- | --- | --- | --- |
|  | **<30** | **30 to 299** | **≥300** |
| **≥90** | low risk | moderate risk | high risk |
| **60-89** | low risk | moderate risk | high risk |
| **45-59** | moderate risk | high risk | very high risk |
| **30-44** | high risk | very high risk | very high risk |
| **15-29** | very high risk | very high risk | very high risk |
| **<15** | very high risk | very high risk | very high risk |

Abbreviations: ACR, albumin-creatinine ratio; CKD, chronic kidney disease; eGFR, estimated glomerular filtration rate; KDIGO, Kidney Disease Improving Global Outcomes.

**Table S2** Definitions of CKM syndrome stages.

| **CKM** **syndrome stages** | **Definition** |
| --- | --- |
| Stage 0 | Normal BMI (<25 kg/m2 or <23 kg/m2 if Asian ethnicity);  Normal waist circumference (<88/102 cm in women/men or <80/90 cm in women/men if Asian ethnicity);  Normoglycemia (fasting blood glucose <100 mg/dL, glycated hemoglobin <5.7%, without self-reported diagnosis of diabetes, without oral hypoglycemic agents or insulin);  Normotension (systolic blood pressure <130 mmHg, diastolic blood pressure <80 mmHg, without self-reported diagnosis of hypertension, without using of antihypertensive medications);  Normal lipid status (high-density lipoprotein cholesterol >50/40 mg/dL in female/male and triglycerides <150 mg/dL);  No evidence of CKD;  No evidence of CVD. |
| Stage 1 | Elevated BMI (≥25 kg/m2 or ≥23 kg/m2 if Asian ethnicity);  Elevated waist circumference (≥88/102 cm in women/men or ≥80/90 cm in women/men if Asian ethnicity);  Prediabetes (fasting blood glucose between 100 mg/dL and 126 mg/dL or glycated hemoglobin between 5.7% and 6.4%). |
| Stage 2 | Elevated fasting serum triglycerides (≥135 mg/dL);  Hypertension (blood pressure ≥140/90 mmHg, self-reported history of hypertension, or current antihypertensive treatment);  Diabetes (fasting blood glucose ≥126 mg/dL, glycated hemoglobin≥6.5%, self-reported history of diabetes, or current hypoglycemic treatment);  Metabolic syndrome (≥3 of the following: elevated waist circumference, high density lipoprotein cholesterol <40 mg/dL for men and <50 mg/dL for women, fasting serum triglycerides ≥150 mg/dL, blood pressure ≥130/80 mmHg and/or current antihypertensive treatment, fasting blood glucose ≥100 mg/dL);  Moderate-to-high-risk CKD^1^. |
| Stage 3 | Very-high-risk CKD^1^;  High predicted 10-year CVD risk (≥20%)^2^. |
| Stage 4 | Self-reported established CVD (coronary heart disease, angina, heart attack, heart failure, and stroke). |

^1^CKD stages were identified based on estimated glomerular filtration rate and urinary albumin-to creatinine ratio (Table S1).

^2^The 10-year CVD risk was calculated using the Framingham risk score.

Abbreviations: BMI, Body Mass Index; CKD, chronic kidney disease; CKM, Cardiovascular-Kidney-Metabolic; CVD, cardiovascular disease.

**Table S3** Comparison of baseline characteristics between participants excluded due to missing B vitamin intake data and those included in the NHANES cohort.

| **Characteristic** | **Excluded participants**  **(N = 3680)** | **Included participants (N = 16829)** | ***p* value** |
| --- | --- | --- | --- |
| **Age (years), mean (SE)** | 51.38(0.63) | 47.88(0.35) | < 0.0001 |
| **Sex, n (%)** |  |  | 0.90 |
| Female | 1924(51.62) | 8660(51.77) |  |
| Male | 1756(48.38) | 8169(48.23) |  |
| **Race or ethnicity, n (%)** |  |  | < 0.0001 |
| Hispanic | 874(21.03) | 3814(13.49) |  |
| Non-Hispanic White | 1045(39.49) | 6753(68.50) |  |
| Non-Hispanic Black | 866(21.37) | 3809(10.24) |  |
| Non-Hispanic Asian | 769(14.33) | 1823(4.59) |  |
| Other | 126(3.78) | 630(3.17) |  |
| **Education level, n (%)** |  |  | < 0.0001 |
| Less than high school | 1065(24.53) | 3167(12.35) |  |
| High school or equivalent | 815(23.25) | 3716(21.79) |  |
| College or above | 1800(52.21) | 9946(65.85) |  |
| **Family PIR, n (%)** |  |  | < 0.0001 |
| <1.0 | 751(17.50) | 3450(13.64) |  |
| 1.0-3.0 | 1855(48.19) | 6896(35.15) |  |
| >3.0 | 1074(34.31) | 6483(51.21) |  |
| **Marital, n (%)** |  |  | < 0.0001 |
| Coupled | 2044(53.67) | 9895(63.44) |  |
| Single or separated | 1636(46.33) | 6934(36.56) |  |
| **Smoking status, n (%)** |  |  | 0.30 |
| Never | 2220(58.55) | 9621(56.74) |  |
| Former | 775(22.99) | 4018(25.28) |  |
| Now | 685(18.46) | 3190(17.98) |  |
| **Alcohol status, n (%)** |  |  | < 0.0001 |
| Non-drinker | 1098(24.18) | 4364(21.39) |  |
| Low to moderate drinker | 2198(69.18) | 9188(58.10) |  |
| Heavy drinker | 384(6.64) | 3277(20.51) |  |
| **Physical activity, n (%)** |  |  | 0.03 |
| Sufficient | 776(24.68) | 4132(28.06) |  |
| Insufficient | 2904(75.32) | 12697(71.94) |  |
| **CKM syndrome stage, n (%)** |  |  | < 0.0001 |
| Stage 0 | 299(3.30) | 2253(15.84) |  |
| Stage 1 | 1298(43.76) | 4582(29.37) |  |
| Stage 2 | 1032(25.05) | 7508(41.14) |  |
| Stage 3 | 579(14.57) | 672(4.87) |  |
| Stage 4 | 472(13.31) | 1814(8.79) |  |

Values are weighted mean (weighted standard errors) for continuous variables or numbers (weighted %) for categorical variables.

Abbreviations: CKM, Cardiovascular-Kidney-Metabolic; NHANES, National Health and Nutrition Examination Survey; PIR, poverty income ratio.

**Table S4** Baseline characteristics of patients with cardiovascular-kidney-metabolic syndrome in the external validation cohort.

| **Characteristic** | **Overall**  **(N = 323)** | **Non-advanced CKM**  **(N = 270)** | **Advanced CKM (N = 53)** | ***p* value** |
| --- | --- | --- | --- | --- |
| **Age (years), mean (SD)** | 51.09(16.47) | 48.01(15.78) | 66.79(9.50) | < 0.0001 |
| **Sex, n (%)** |  |  |  | 0.048 |
| Female | 164(50.77) | 144(53.33) | 20(37.74) |  |
| Male | 159(49.23) | 126(46.67) | 33(62.26) |  |
| **Marital, n (%)** |  |  |  | 0.621 |
| Coupled | 198(61.30) | 165(61.11) | 33(62.26) |  |
| Single or separated | 125(38.70) | 105(38.89) | 20(37.74) |  |
| **Vitamin B1 (mg), mean (SD)** | 1.43(0.77) | 1.39(0.72) | 1.61(0.95) | 0.18 |
| **Vitamin B2 (mg), mean (SD)** | 2.05(1.47) | 2.08(0.93) | 1.93(1.55) | 0.02 |
| **Vitamin B6 (mg), mean (SD)** | 2.10(2.13) | 2.13(1.73) | 2.02(2.20) | 0.01 |
| **Vitamin B12 (mg), mean (SD)** | 4.50(5.82) | 4.45(6.15) | 4.77(3.72) | 0.12 |
| **Niacin (mg), mean (SD)** | 24.75(19.46) | 24.87(20.47) | 24.13(13.31) | 0.03 |
| **Total folate (μg), mean (SD)** | 353.89(207.35) | 351.59(205.31) | 365.62(219.12) | 0.14 |

Values are mean (standard deviation) for continuous variables or numbers (%) for categorical variables.

Abbreviations: CKM, Cardiovascular-Kidney-Metabolic; PIR, poverty income ratio.

**Table S5** Association between continuous B vitamin intakes and advanced CKM syndrome in the external validation cohort.

|  | **Vitamin levels** | | | | | |  |
| --- | --- | --- | --- | --- | --- | --- | --- |
|  | **Q1** | **Q2** | **Q3** | **Q4** | **Per 1 unit**  **increment** | ***P* for trend** | |
| **Vitamin B1** | 1 (reference) | 1.51(0.61,3.76) | 1.84(0.58,5.79) | 2.38(0.72,7.92) | 0.94(0.85,1.07) | 0.10 | |
| **Vitamin B2** | 1 (reference) | 0.96(0.79,1.18) | 0.82(0.63,0.96) | 0.76(0.68,0.97) | 0.86(0.80,0.92) | 0.01 | |
| **Vitamin B6** | 1 (reference) | 0.95(0.82,1.14) | 0.84(0.69,1.06) | 0.78(0.68,0.96) | 0.90(0.85,0.97) | 0.04 | |
| **Vitamin B12** | 1 (reference) | 0.86(0.68,1.10) | 1.02(0.86,1.20) | 0.86(0.74,1.06) | 0.98(0.96,1.00) | 0.18 | |
| **Niacin** | 1 (reference) | 1.04(0.90,1.22) | 0.86(0.72,1.05) | 0.92(0.68,1.14) | 0.99(0.98,0.99) | 0.13 | |
| **Total folate** | 1 (reference) | 0.95(0.76,1.13) | 0.84(0.70,1.04) | 0.82(0.69,0.98) | 1.00(1.00,1.00) | 0.02 | |

Values are OR (95% CI).

Model was adjusted for age, sex, and marital status.

Abbreviations: CI, Confidence interval; CKM, Cardiovascular-Kidney-Metabolic; OR, Odds ratios.

**Table S6** Age- and sex-stratified associations of dietary vitamin B1 intake with advanced CKM syndrome.

|  | **Quartiles of Vitamin B1 intakes** | | | | | | **Continuous vitamin B1 intakes** | | |
| --- | --- | --- | --- | --- | --- | --- | --- | --- | --- |
|  | **Q1** | **Q2** | **Q3** | **Q4** | ***p interaction*** |  | | **Per 1 unit increment** | ***p interaction*** |
| **Age** |  |  |  |  | 0.080 |  | |  | 0.160 |
| 20-44 | 1(reference) | 1.19(0.64,2.19) | 0.79(0.44,1.42) | 0.63(0.35,1.11) |  |  | | 0.75(0.56,1.01) |  |
| 45-64 | 1(reference) | 1.17(0.81,1.69) | 0.86(0.57,1.29) | 1.01(0.70,1.44) |  |  | | 0.95(0.82,1.10) |  |
| ≥65 | 1(reference) | 1.05(0.84,1.31) | 0.74(0.57,0.97) | 0.90(0.67,1.21) |  |  | | 0.92(0.80,1.06) |  |
| **Sex** |  |  |  |  | 0.468 |  | |  | 0.155 |
| Female | 1(reference) | 1.05(0.85,1.31) | 0.74(0.58,0.95) | 0.92(0.71,1.21) |  |  | | 0.86(0.74,1.01) |  |
| Male | 1(reference) | 1.21(0.90,1.63) | 0.84(0.62,1.14) | 0.97(0.72,1.30) |  |  | | 0.94(0.83,1.07) |  |

Values are weighted OR (95% CI). Model: each stratification was adjusted for age, sex, ethnicity, educational level, family income, marital status, smoking status, alcohol status, physical activity, and HEI-2015 except the stratification factor itself.

Abbreviations: CI, Confidence interval; CKM, Cardiovascular-Kidney-Metabolic; HEI-2015, Healthy Eating Index-2015; OR, Odds ratios.

**Table S7** Age- and sex-stratified associations of dietary vitamin B2 intake with advanced CKM syndrome.

|  | **Quartiles of Vitamin B2 intakes** | | | | | | **Continuous vitamin B2 intakes** | | |
| --- | --- | --- | --- | --- | --- | --- | --- | --- | --- |
|  | **Q1** | **Q2** | **Q3** | **Q4** | ***p interaction*** |  | | **Per 1 unit increment** | ***p interaction*** |
| **Age** |  |  |  |  | 0.780 |  | |  | 0.442 |
| 20-44 | 1(reference) | 1.30(0.80,2.13) | 0.85(0.46,1.57) | 0.76(0.42,1.38) |  |  | | 0.82(0.70,0.96) |  |
| 45-64 | 1(reference) | 0.82(0.60,1.11) | 0.67(0.48,0.92) | 0.62(0.46,0.85) |  |  | | 0.87(0.77,0.98) |  |
| ≥65 | 1(reference) | 1.19(0.90,1.58) | 0.91(0.67,1.23) | 0.86(0.64,1.15) |  |  | | 0.88(0.80,0.97) |  |
| **Sex** |  |  |  |  | 0.204 |  | |  | 0.131 |
| Female | 1(reference) | 1.02(0.83,1.25) | 0.71(0.55,0.92) | 0.63(0.47,0.85) |  |  | | 0.80(0.70,0.91) |  |
| Male | 1(reference) | 1.18(0.80,1.72) | 0.94(0.68,1.29) | 0.91(0.64,1.30) |  |  | | 0.90(0.82,0.99) |  |

Values are weighted OR (95% CI). Model: each stratification was adjusted for age, sex, ethnicity, educational level, family income, marital status, smoking status, alcohol status, physical activity, and HEI-2015 except the stratification factor itself.

Abbreviations: CI, Confidence interval; CKM, Cardiovascular-Kidney-Metabolic; HEI-2015, Healthy Eating Index-2015; OR, Odds ratios.

**Table S8** Age- and sex-stratified associations of dietary vitamin B6 intake with advanced CKM syndrome.

|  | **Quartiles of Vitamin B6 intakes** | | | | | | **Continuous vitamin B6 intakes** | | |
| --- | --- | --- | --- | --- | --- | --- | --- | --- | --- |
|  | **Q1** | **Q2** | **Q3** | **Q4** | ***p interaction*** |  | | **Per 1 unit increment** | ***p interaction*** |
| **Age** |  |  |  |  | 0.106 |  | |  | 0.342 |
| 20-44 | 1(reference) | 0.61(0.35,1.07) | 0.42(0.22,0.78) | 0.39(0.23,0.67) |  |  | | 0.91(0.85,1.02) |  |
| 45-64 | 1(reference) | 0.92(0.66,1.27) | 0.70(0.48,1.03) | 0.73(0.51,1.06) |  |  | | 0.89(0.82,0.94) |  |
| ≥65 | 1(reference) | 1.02(0.78,1.33) | 1.09(0.87,1.36) | 1.16(0.90,1.51) |  |  | | 0.84(0.78,0.90) |  |
| **Sex** |  |  |  |  | 0.192 |  | |  | 0.867 |
| Female | 1(reference) | 0.89(0.73,1.08) | 0.65(0.51,0.82) | 0.60(0.43,0.83) |  |  | | 0.90(0.85,0.97) |  |
| Male | 1(reference) | 0.95(0.69,1.31) | 0.97(0.70,1.33) | 0.82(0.61,1.11) |  |  | | 0.86(0.81,0.89) |  |

Values are weighted OR (95% CI). Model: each stratification was adjusted for age, sex, ethnicity, educational level, family income, marital status, smoking status, alcohol status, physical activity, and HEI-2015 except the stratification factor itself.

Abbreviations: CI, Confidence interval; CKM, Cardiovascular-Kidney-Metabolic; HEI-2015, Healthy Eating Index-2015; OR, Odds ratios.

**Table S9** Age- and sex-stratified associations of dietary vitamin B12 intake with advanced CKM syndrome.

|  | **Quartiles of Vitamin B12 intakes** | | | | | | **Continuous vitamin B12 intakes** | | |
| --- | --- | --- | --- | --- | --- | --- | --- | --- | --- |
|  | **Q1** | **Q2** | **Q3** | **Q4** | ***p interaction*** |  | | **Per 1 unit increment** | ***p interaction*** |
| **Age** |  |  |  |  | 0.174 |  | |  | 0.749 |
| 20-44 | 1(reference) | 0.80(0.50,1.29) | 0.97(0.59,1.61) | 1.14(0.75,1.74) |  |  | | 1.01(0.98,1.05) |  |
| 45-64 | 1(reference) | 0.77(0.55,1.10) | 0.99(0.69,1.43) | 0.62(0.43,0.88) |  |  | | 0.98(0.94,1.02) |  |
| ≥65 | 1(reference) | 0.88(0.64,1.20) | 0.86(0.65,1.16) | 0.89(0.70,1.15) |  |  | | 0.98(0.97,1.00) |  |
| **Sex** |  |  |  |  | 0.123 |  | |  | 0.411 |
| Female | 1(reference) | 0.92(0.73,1.18) | 0.88(0.67,1.14) | 0.84(0.64,1.11) |  |  | | 0.98(0.96,1.01) |  |
| Male | 1(reference) | 0.70(0.49,1.01) | 1.08(0.84,1.38) | 0.80(0.61,1.06) |  |  | | 0.99(0.98,1.00) |  |

Values are weighted OR (95% CI). Model: each stratification was adjusted for age, sex, ethnicity, educational level, family income, marital status, smoking status, alcohol status, physical activity, and HEI-2015 except the stratification factor itself.

Abbreviations: CI, Confidence interval; CKM, Cardiovascular-Kidney-Metabolic; HEI-2015, Healthy Eating Index-2015; OR, Odds ratios.

**Table S10** Age- and sex-stratified associations of dietary niacin intake with advanced CKM syndrome.

|  | **Quartiles of niacin intakes** | | | | | | **Continuous niacin intakes** | | |
| --- | --- | --- | --- | --- | --- | --- | --- | --- | --- |
|  | **Q1** | **Q2** | **Q3** | **Q4** | ***p interaction*** |  | | **Per 1 unit increment** | ***p interaction*** |
| **Age** |  |  |  |  | 0.114 |  | |  | 0.348 |
| 20-44 | 1(reference) | 1.16(0.71,1.88) | 0.58(0.31,1.05) | 0.59(0.34,1.03) |  |  | | 0.99(0.97,1.01) |  |
| 45-64 | 1(reference) | 0.89(0.65,1.21) | 0.80(0.56,1.15) | 0.79(0.55,1.12) |  |  | | 0.99(0.98,1.00) |  |
| ≥65 | 1(reference) | 1.02(0.78,1.33) | 0.91(0.73,1.13) | 0.92(0.65,1.30) |  |  | | 0.99(0.98,1.00) |  |
| **Sex** |  |  |  |  | 0.753 |  | |  | 0.121 |
| Female | 1(reference) | 1.01(0.84,1.21) | 0.81(0.64,1.03) | 0.92(0.67,1.27) |  |  | | 1.00(0.99,1.01) |  |
| Male | 1(reference) | 0.97(0.70,1.34) | 0.83(0.62,1.11) | 0.80(0.57,1.12) |  |  | | 0.99(0.98,1.00) |  |

Values are weighted OR (95% CI). Model: each stratification was adjusted for age, sex, ethnicity, educational level, family income, marital status, smoking status, alcohol status, physical activity, and HEI-2015 except the stratification factor itself.

Abbreviations: CI, Confidence interval; CKM, Cardiovascular-Kidney-Metabolic; HEI-2015, Healthy Eating Index-2015; OR, Odds ratios.

**Table S11** Age- and sex-stratified associations of dietary total folate intake with advanced CKM syndrome.

|  | **Quartiles of total folate intakes** | | | | | | **Continuous total folate intakes** | | |
| --- | --- | --- | --- | --- | --- | --- | --- | --- | --- |
|  | **Q1** | **Q2** | **Q3** | **Q4** | ***p interaction*** |  | | **Per 1 unit increment** | ***p interaction*** |
| **Age** |  |  |  |  | 0.346 |  | |  | 0.456 |
| 20-44 | 1(reference) | 0.83(0.47,1.46) | 0.83(0.48,1.43) | 0.39(0.21,0.74) |  |  | | 1.00(1.00,1.00) |  |
| 45-64 | 1(reference) | 0.85(0.64,1.14) | 1.00(0.69,1.45) | 0.84(0.58,1.22) |  |  | | 1.00(1.00,1.00) |  |
| ≥65 | 1(reference) | 0.94(0.72,1.22) | 0.75(0.60,0.95) | 0.83(0.67,1.05) |  |  | | 1.00(1.00,1.00) |  |
| **Sex** |  |  |  |  | 0.106 |  | |  | 0.298 |
| Female | 1(reference) | 0.91(0.72,1.15) | 0.81(0.62,1.05) | 0.66(0.49,0.88) |  |  | | 1.00(1.00,1.00) |  |
| Male | 1(reference) | 0.90(0.62,1.30) | 0.86(0.63,1.16) | 0.88(0.63,1.23) |  |  | | 1.00(1.00,1.00) |  |

Values are weighted OR (95% CI). Model: each stratification was adjusted for age, sex, ethnicity, educational level, family income, marital status, smoking status, alcohol status, physical activity, and HEI-2015 except the stratification factor itself.

Abbreviations: CI, Confidence interval; CKM, Cardiovascular-Kidney-Metabolic; HEI-2015, Healthy Eating Index-2015; OR, Odds ratios.

**Table S12** Association between continuous B vitamin intakes and advanced CKM syndrome after excluding participants with baseline self-reported history of cancer.

| **Vitamin B** | Model 1 | |  | Model 2 | |  | Model 3 | |
| --- | --- | --- | --- | --- | --- | --- | --- | --- |
|  | OR (95% CI) | *p* value |  | OR (95% CI) | *p* value |  | OR (95% CI) | *p* value |
| **Vitamin B1** |  |  | |  |  | |  |  |
| Per 1 unit increment | 0.90(0.82,0.98) | 0.02 | | 0.92(0.84,1.01) | 0.07 | | 0.92(0.84,1.01) | 0.08 |
| **Vitamin B2** |  |  | |  |  | |  |  |
| Per 1 unit increment | 0.88(0.82,0.94) | <0.001 | | 0.90(0.84,0.96) | 0.002 | | 0.88(0.83,0.94) | <0.001 |
| **Vitamin B6** |  |  | |  |  | |  |  |
| Per 1 unit increment | 0.88(0.83,0.93) | <0.0001 | | 0.85(0.79,0.91) | <0.0001 | | 0.87(0.82,0.92) | <0.0001 |
| **Vitamin B12** |  |  | |  |  | |  |  |
| Per 1 unit increment | 0.99(0.96,1.01) | 0.23 | | 0.99(0.97,1.01) | 0.26 | | 0.99(0.97,1.01) | 0.18 |
| **Niacin** |  |  | |  |  | |  |  |
| Per 1 unit increment | 0.99(0.98,1.00) | 0.01 | | 0.99(0.99,1.00) | 0.04 | | 0.99(0.99,1.00) | 0.04 |
| **Total folate** |  |  | |  |  | |  |  |
| Per 1 unit increment | 1.00(1.00,1.00) | 0.09 | | 1.00(1.00,1.00) | 0.25 | | 1.00(1.00,1.00) | 0.38 |

Values are weighted OR (95% CI).

Model 1: adjusted for age, sex, and ethnicity.

Model 2: Model 1 + educational level, family income, and marital status.

Model 3: Model 2 + smoking status, alcohol status, physical activity, and HEI-2015.

Abbreviations: CI, Confidence interval; CKM, Cardiovascular-Kidney-Metabolic; HEI-2015, Healthy Eating Index-2015; OR, Odds ratios.

**Table S13** Association between quartiles of B vitamin intakes and advanced CKM syndrome after excluding participants with baseline self-reported history of cancer.

| **Vitamin B** | Model 1 | |  | Model 2 | |  | Model 3 | |
| --- | --- | --- | --- | --- | --- | --- | --- | --- |
|  | OR (95% CI) | *p* value |  | OR (95% CI) | *p* value |  | OR (95% CI) | *p* value |
| **Vitamin B1** |  |  | |  |  | |  |  |
| Q1 | 1 (reference) |  | | 1 (reference) |  | | 1 (reference) |  |
| Q2 | 0.99(0.78,1.25) | 0.92 | | 1.04(0.82,1.32) | 0.75 | | 1.08(0.85,1.37) | 0.51 |
| Q3 | 0.75(0.63,0.90) | 0.002 | | 0.80(0.67,0.96) | 0.02 | | 0.82(0.68,0.99) | 0.04 |
| Q4 | 0.83(0.66,1.05) | 0.11 | | 0.90(0.72,1.14) | 0.40 | | 0.92(0.72,1.17) | 0.49 |
| *P* for the trend | 0.01 |  | | 0.09 |  | | 0.13 |  |
| **Vitamin B2** |  |  | |  |  | |  |  |
| Q1 | 1 (reference) |  | | 1 (reference) |  | | 1 (reference) |  |
| Q2 | 1.05(0.86,1.28) | 0.62 | | 1.14(0.94,1.39) | 0.19 | | 1.13(0.92,1.38) | 0.23 |
| Q3 | 0.73(0.62,0.87) | <0.001 | | 0.85(0.71,1.02) | 0.08 | | 0.85(0.70,1.02) | 0.09 |
| Q4 | 0.72(0.59,0.87) | 0.001 | | 0.82(0.67,1.01) | 0.06 | | 0.79(0.63,0.99) | 0.04 |
| *P* for the trend | <0.0001 |  | | 0.004 |  | | 0.003 |  |
| **Vitamin B6** |  |  | |  |  | |  |  |
| Q1 | 1 (reference) |  | | 1 (reference) |  | | 1 (reference) |  |
| Q2 | 0.82(0.67,0.99) | 0.04 | | 0.82(0.68,1.00) | 0.05 | | 0.90(0.74,1.10) | 0.30 |
| Q3 | 0.64(0.52,0.78) | <0.0001 | | 0.65(0.53,0.79) | <0.0001 | | 0.76(0.60,0.96) | 0.02 |
| Q4 | 0.64(0.51,0.80) | <0.001 | | 0.64(0.52,0.80) | <0.001 | | 0.75(0.59,0.95) | 0.02 |
| *P* for the trend | <0.0001 |  | | <0.0001 |  | | 0.01 |  |
| **Vitamin B12** |  |  | |  |  | |  |  |
| Q1 | 1 (reference) |  | | 1 (reference) |  | | 1 (reference) |  |
| Q2 | 0.75(0.59,0.94) | 0.01 | | 0.79(0.63,1.00) | 0.05 | | 0.82(0.65,1.03) | 0.09 |
| Q3 | 0.88(0.72,1.08) | 0.23 | | 0.95(0.76,1.19) | 0.65 | | 0.97(0.77,1.22) | 0.77 |
| Q4 | 0.69(0.56,0.84) | <0.001 | | 0.74(0.61,0.91) | 0.004 | | 0.76(0.62,0.93) | 0.01 |
| *P* for the trend | 0.002 |  | | 0.02 |  | | 0.04 |  |
| **Niacin** |  |  | |  |  | |  |  |
| Q1 | 1 (reference) |  | | 1 (reference) |  | | 1 (reference) |  |
| Q2 | 0.90(0.77,1.05) | 0.18 | | 0.97(0.83,1.14) | 0.71 | | 0.98(0.83,1.15) | 0.77 |
| Q3 | 0.77(0.63,0.93) | 0.01 | | 0.85(0.69,1.03) | 0.09 | | 0.86(0.71,1.06) | 0.15 |
| Q4 | 0.71(0.54,0.94) | 0.02 | | 0.80(0.60,1.07) | 0.14 | | 0.82(0.61,1.11) | 0.19 |
| *P* for the trend | 0.004 |  | | 0.05 |  | | 0.09 |  |
| **Total folate** |  |  | |  |  | |  |  |
| Q1 | 1 (reference) |  | | 1 (reference) |  | | 1 (reference) |  |
| Q2 | 0.80(0.64,1.00) | 0.05 | | 0.85(0.67,1.07) | 0.16 | | 0.89(0.70,1.13) | 0.32 |
| Q3 | 0.72(0.59,0.89) | 0.002 | | 0.79(0.63,0.98) | 0.03 | | 0.82(0.66,1.03) | 0.09 |
| Q4 | 0.64(0.51,0.79) | <0.0001 | | 0.71(0.56,0.91) | 0.01 | | 0.76(0.60,0.97) | 0.03 |
| *P* for the trend | <0.0001 |  | | 0.005 |  | | 0.02 |  |

Values are weighted OR (95% CI).

Model 1: adjusted for age, sex, and ethnicity.

Model 2: Model 1 + educational level, family income, and marital status.

Model 3: Model 2 + smoking status, alcohol status, physical activity, and HEI-2015.

Abbreviations: CI, Confidence interval; CKM, Cardiovascular-Kidney-Metabolic; HEI-2015, Healthy Eating Index-2015; OR, Odds ratios.

**Table S14** Association between continuous B vitamin intakes and advanced CKM syndrome after iterative imputation on covariates.

| **Vitamin B** | Model 1 | |  | Model 2 | |  | Model 3 | |
| --- | --- | --- | --- | --- | --- | --- | --- | --- |
|  | OR (95% CI) | *p* value |  | OR (95% CI) | *p* value |  | OR (95% CI) | *p* value |
| **Vitamin B1** |  |  | |  |  | |  |  |
| Per 1 unit increment | 0.89(0.83,0.95) | <0.001 | | 0.90(0.84,0.96) | 0.002 | | 0.90(0.84,0.97) | 0.004 |
| **Vitamin B2** |  |  | |  |  | |  |  |
| Per 1 unit increment | 0.88(0.83,0.93) | <0.0001 | | 0.89(0.85,0.94) | <0.0001 | | 0.88(0.83,0.93) | <0.0001 |
| **Vitamin B6** |  |  | |  |  | |  |  |
| Per 1 unit increment | 0.91(0.86,0.96) | <0.001 | | 0.87(0.81,0.93) | <0.0001 | | 0.88(0.83,0.94) | <0.001 |
| **Vitamin B12** |  |  | |  |  | |  |  |
| Per 1 unit increment | 0.99(0.97,1.01) | 0.15 | | 0.99(0.97,1.01) | 0.18 | | 0.99(0.97,1.00) | 0.15 |
| **Niacin** |  |  | |  |  | |  |  |
| Per 1 unit increment | 0.99(0.99,1.00) | 0.003 | | 0.99(0.99,1.00) | 0.005 | | 0.99(0.99,1.00) | 0.003 |
| **Total folate** |  |  | |  |  | |  |  |
| Per 1 unit increment | 1.00(1.00,1.00) | 0.01 | | 1.00(1.00,1.00) | 0.03 | | 1.00(1.00,1.00) | 0.10 |

Values are weighted OR (95% CI).

Model 1: adjusted for age, sex, and ethnicity.

Model 2: Model 1 + educational level, family income, and marital status.

Model 3: Model 2 + smoking status, alcohol status, physical activity, and HEI-2015.

Abbreviations: CI, Confidence interval; CKM, Cardiovascular-Kidney-Metabolic; HEI-2015, Healthy Eating Index-2015; OR, Odds ratios.

**Table S15** Association between quartiles of B vitamin intakes and advanced CKM syndrome after iterative imputation on covariates.

| **Vitamin B** | Model 1 | |  | Model 2 | |  | Model 3 | |
| --- | --- | --- | --- | --- | --- | --- | --- | --- |
|  | OR (95% CI) | *p* value |  | OR (95% CI) | *p* value |  | OR (95% CI) | *p* value |
| **Vitamin B1** |  |  | |  |  | |  |  |
| Q1 | 1 (reference) |  | | 1 (reference) |  | | 1 (reference) |  |
| Q2 | 0.90(0.78,1.05) | 0.18 | | 0.96(0.82,1.11) | 0.55 | | 1.00(0.86,1.16) | 0.97 |
| Q3 | 0.73(0.62,0.85) | <0.001 | | 0.77(0.66,0.90) | 0.002 | | 0.80(0.68,0.94) | 0.01 |
| Q4 | 0.76(0.65,0.89) | <0.001 | | 0.82(0.70,0.95) | 0.01 | | 0.84(0.72,0.98) | 0.03 |
| *P* for the trend | <0.0001 |  | | <0.001 |  | | 0.003 |  |
| **Vitamin B2** |  |  | |  |  | |  |  |
| Q1 | 1 (reference) |  | | 1 (reference) |  | | 1 (reference) |  |
| Q2 | 0.91(0.78,1.06) | 0.22 | | 0.97(0.83,1.14) | 0.71 | | 0.98(0.83,1.14) | 0.75 |
| Q3 | 0.66(0.58,0.74) | <0.0001 | | 0.73(0.64,0.83) | <0.0001 | | 0.74(0.64,0.84) | <0.0001 |
| Q4 | 0.65(0.56,0.75) | <0.0001 | | 0.72(0.62,0.83) | <0.0001 | | 0.70(0.59,0.82) | <0.0001 |
| *P* for the trend | <0.0001 |  | | <0.0001 |  | | <0.0001 |  |
| **Vitamin B6** |  |  | |  |  | |  |  |
| Q1 | 1 (reference) |  | | 1 (reference) |  | | 1 (reference) |  |
| Q2 | 0.87(0.75,1.00) | 0.05 | | 0.87(0.76,1.00) | 0.06 | | 0.96(0.83,1.11) | 0.54 |
| Q3 | 0.69(0.60,0.80) | <0.0001 | | 0.70(0.60,0.80) | <0.0001 | | 0.80(0.68,0.95) | 0.01 |
| Q4 | 0.63(0.53,0.75) | <0.0001 | | 0.63(0.53,0.75) | <0.0001 | | 0.72(0.59,0.87) | 0.001 |
| *P* for the trend | <0.0001 |  | | <0.0001 |  | | <0.001 |  |
| **Vitamin B12** |  |  | |  |  | |  |  |
| Q1 | 1 (reference) |  | | 1 (reference) |  | | 1 (reference) |  |
| Q2 | 0.76(0.62,0.93) | 0.01 | | 0.80(0.66,0.98) | 0.03 | | 0.84(0.69,1.02) | 0.08 |
| Q3 | 0.88(0.77,1.01) | 0.07 | | 0.94(0.81,1.08) | 0.37 | | 0.96(0.83,1.12) | 0.63 |
| Q4 | 0.70(0.61,0.81) | <0.0001 | | 0.75(0.65,0.86) | <0.001 | | 0.76(0.66,0.89) | <0.001 |
| *P* for the trend | <0.0001 |  | | 0.002 |  | | 0.01 |  |
| **Niacin** |  |  | |  |  | |  |  |
| Q1 | 1 (reference) |  | | 1 (reference) |  | | 1 (reference) |  |
| Q2 | 0.82(0.73,0.92) | <0.001 | | 0.87(0.77,0.97) | 0.02 | | 0.89(0.79,1.00) | 0.05 |
| Q3 | 0.72(0.63,0.82) | <0.0001 | | 0.77(0.67,0.88) | <0.001 | | 0.80(0.69,0.91) | 0.002 |
| Q4 | 0.70(0.57,0.84) | <0.001 | | 0.75(0.62,0.92) | 0.01 | | 0.77(0.63,0.94) | 0.01 |
| *P* for the trend | <0.0001 |  | | 0.002 |  | | 0.004 |  |
| **Total folate** |  |  | |  |  | |  |  |
| Q1 | 1 (reference) |  | | 1 (reference) |  | | 1 (reference) |  |
| Q2 | 0.78(0.67,0.91) | 0.002 | | 0.82(0.70,0.96) | 0.01 | | 0.86(0.73,1.01) | 0.07 |
| Q3 | 0.67(0.58,0.78) | <0.0001 | | 0.72(0.62,0.84) | <0.0001 | | 0.75(0.64,0.89) | 0.001 |
| Q4 | 0.64(0.54,0.75) | <0.0001 | | 0.70(0.59,0.82) | <0.0001 | | 0.75(0.63,0.89) | 0.002 |
| *P* for the trend | <0.0001 |  | | <0.0001 |  | | <0.001 |  |

Values are weighted OR (95% CI).

Model 1: adjusted for age, sex, and ethnicity.

Model 2: Model 1 + educational level, family income, and marital status.

Model 3: Model 2 + smoking status, alcohol status, physical activity, and HEI-2015.

Abbreviations: CI, Confidence interval; CKM, Cardiovascular-Kidney-Metabolic; HEI-2015, Healthy Eating Index-2015; OR, Odds ratios.

**Table S16** Association between continuous B vitamin intakes and advanced CKM syndrome after excluding participants taking potential CKM syndrome medications^a^

| **Vitamin B** | Model 1 | |  | Model 2 | |  | Model 3 | |
| --- | --- | --- | --- | --- | --- | --- | --- | --- |
|  | OR (95% CI) | *p* value |  | OR (95% CI) | *p* value |  | OR (95% CI) | *p* value |
| **Vitamin B1** |  |  | |  |  | |  |  |
| Per 1 unit increment | 0.90(0.84,0.97) | 0.01 | | 0.92(0.85,0.99) | 0.03 | | 0.92(0.85,1.00) | 0.05 |
| **Vitamin B2** |  |  | |  |  | |  |  |
| Per 1 unit increment | 0.87(0.82,0.92) | <0.0001 | | 0.89(0.84,0.94) | <0.001 | | 0.87(0.82,0.93) | <0.0001 |
| **Vitamin B6** |  |  | |  |  | |  |  |
| Per 1 unit increment | 0.89(0.83,0.95) | <0.001 | | 0.85(0.79,0.91) | <0.0001 | | 0.87(0.81,0.92) | <0.0001 |
| **Vitamin B12** |  |  | |  |  | |  |  |
| Per 1 unit increment | 0.99(0.97,1.01) | 0.15 | | 0.99(0.97,1.01) | 0.22 | | 0.99(0.98,1.00) | 0.18 |
| **Niacin** |  |  | |  |  | |  |  |
| Per 1 unit increment | 0.99(0.98,1.00) | 0.001 | | 0.99(0.98,1.00) | 0.01 | | 0.99(0.98,1.00) | 0.01 |
| **Total folate** |  |  | |  |  | |  |  |
| Per 1 unit increment | 1.00(1.00,1.00) | 0.01 | | 1.00(1.00,1.00) | 0.04 | | 1.00(1.00,1.00) | 0.10 |

Values are weighted OR (95% CI).

Model 1: adjusted for age, sex, and ethnicity.

Model 2: Model 1 + educational level, family income, and marital status.

Model 3: Model 2 + smoking status, alcohol status, physical activity, and HEI-2015.

^a^Including sodium-glucose cotransporter-2 inhibitors and glucagon-like peptide 1 receptor agonists.

Abbreviations: CI, Confidence interval; CKM, Cardiovascular-Kidney-Metabolic; HEI-2015, Healthy Eating Index-2015; OR, Odds ratios.

**Table S17** Association between quartiles of B vitamin intakes and advanced CKM syndrome after excluding participants taking potential CKM syndrome medications^a^

| **Vitamin B** | Model 1 | |  | Model 2 | |  | Model 3 | |
| --- | --- | --- | --- | --- | --- | --- | --- | --- |
|  | OR (95% CI) | *p* value |  | OR (95% CI) | *p* value |  | OR (95% CI) | *p* value |
| **Vitamin B1** |  |  | |  |  | |  |  |
| Q1 | 1 (reference) |  | | 1 (reference) |  | | 1 (reference) |  |
| Q2 | 0.94(0.79,1.12) | 0.48 | | 1.00(0.84,1.18) | 0.96 | | 1.04(0.87,1.23) | 0.67 |
| Q3 | 0.68(0.58,0.81) | <0.0001 | | 0.73(0.62,0.86) | <0.001 | | 0.75(0.64,0.89) | 0.001 |
| Q4 | 0.82(0.68,0.98) | 0.03 | | 0.88(0.73,1.05) | 0.15 | | 0.89(0.74,1.08) | 0.24 |
| *P* for the trend | 0.001 |  | | 0.01 |  | | 0.02 |  |
| **Vitamin B2** |  |  | |  |  | |  |  |
| Q1 | 1 (reference) |  | | 1 (reference) |  | | 1 (reference) |  |
| Q2 | 0.90(0.76,1.07) | 0.22 | | 0.97(0.82,1.15) | 0.72 | | 0.97(0.82,1.16) | 0.76 |
| Q3 | 0.67(0.57,0.78) | <0.0001 | | 0.77(0.66,0.91) | 0.002 | | 0.77(0.65,0.91) | 0.003 |
| Q4 | 0.65(0.54,0.78) | <0.0001 | | 0.72(0.60,0.87) | <0.001 | | 0.70(0.57,0.86) | 0.001 |
| *P* for the trend | <0.0001 |  | | <0.0001 |  | | <0.0001 |  |
| **Vitamin B6** |  |  | |  |  | |  |  |
| Q1 | 1 (reference) |  | | 1 (reference) |  | | 1 (reference) |  |
| Q2 | 0.84(0.71,0.98) | 0.03 | | 0.84(0.72,0.99) | 0.03 | | 0.93(0.79,1.10) | 0.39 |
| Q3 | 0.64(0.54,0.76) | <0.0001 | | 0.65(0.55,0.77) | <0.0001 | | 0.76(0.63,0.93) | 0.01 |
| Q4 | 0.61(0.51,0.73) | <0.0001 | | 0.61(0.51,0.74) | <0.0001 | | 0.71(0.58,0.87) | 0.001 |
| *P* for the trend | <0.0001 |  | | <0.0001 |  | | <0.001 |  |
| **Vitamin B12** |  |  | |  |  | |  |  |
| Q1 | 1 (reference) |  | | 1 (reference) |  | | 1 (reference) |  |
| Q2 | 0.75(0.61,0.92) | 0.01 | | 0.80(0.65,0.98) | 0.04 | | 0.83(0.68,1.03) | 0.08 |
| Q3 | 0.86(0.73,1.02) | 0.09 | | 0.93(0.78,1.11) | 0.41 | | 0.96(0.80,1.15) | 0.62 |
| Q4 | 0.69(0.58,0.82) | <0.0001 | | 0.74(0.63,0.88) | <0.001 | | 0.76(0.64,0.90) | 0.002 |
| *P* for the trend | <0.0001 |  | | 0.01 |  | | 0.02 |  |
| **Niacin** |  |  | |  |  | |  |  |
| Q1 | 1 (reference) |  | | 1 (reference) |  | | 1 (reference) |  |
| Q2 | 0.91(0.81,1.01) | 0.08 | | 0.98(0.87,1.10) | 0.68 | | 1.00(0.89,1.13) | 0.98 |
| Q3 | 0.73(0.62,0.85) | <0.001 | | 0.80(0.68,0.93) | 0.005 | | 0.81(0.69,0.95) | 0.01 |
| Q4 | 0.68(0.54,0.86) | 0.001 | | 0.76(0.60,0.96) | 0.02 | | 0.78(0.61,0.99) | 0.04 |
| *P* for the trend | <0.001 |  | | 0.004 |  | | 0.01 |  |
| **Total folate** |  |  | |  |  | |  |  |
| Q1 | 1 (reference) |  | | 1 (reference) |  | | 1 (reference) |  |
| Q2 | 0.78(0.66,0.93) | 0.005 | | 0.83(0.69,0.99) | 0.04 | | 0.87(0.72,1.04) | 0.12 |
| Q3 | 0.65(0.55,0.77) | <0.0001 | | 0.70(0.59,0.84) | <0.001 | | 0.73(0.61,0.89) | 0.002 |
| Q4 | 0.64(0.54,0.77) | <0.0001 | | 0.72(0.59,0.87) | <0.001 | | 0.77(0.63,0.94) | 0.01 |
| *P* for the trend | <0.0001 |  | | <0.001 |  | | 0.003 |  |

Values are weighted OR (95% CI).

Model 1: adjusted for age, sex, and ethnicity.

Model 2: Model 1 + educational level, family income, and marital status.

Model 3: Model 2 + smoking status, alcohol status, physical activity, and HEI-2015.

^a^Including sodium-glucose cotransporter-2 inhibitors and glucagon-like peptide 1 receptor agonists.

Abbreviations: CI, Confidence interval; CKM, Cardiovascular-Kidney-Metabolic; HEI-2015, Healthy Eating Index-2015; OR, Odds ratios.

**Table S18** Association between continuous B vitamin intakes and advanced CKM syndrome by ordinal logistic regression models.

| **Vitamin B** | Model 1 | |  | Model 2 | |  | Model 3 | |
| --- | --- | --- | --- | --- | --- | --- | --- | --- |
|  | OR (95% CI) | *p* value |  | OR (95% CI) | *p* value |  | OR (95% CI) | *p* value |
| **Vitamin B1** |  |  | |  |  | |  |  |
| Per 1 unit increment | 0.91(0.86,0.98) | 0.01 | | 0.93(0.87,0.99) | 0.02 | | 0.95(0.89,1.02) | 0.16 |
| **Vitamin B2** |  |  | |  |  | |  |  |
| Per 1 unit increment | 0.88(0.82,0.93) | <0.0001 | | 0.91(0.84,0.95) | <0.001 | | 0.91(0.82,0.95) | <0.001 |
| **Vitamin B6** |  |  | |  |  | |  |  |
| Per 1 unit increment | 0.90(0.86,0.98) | 0.003 | | 0.89(0.82,0.96) | <0.001 | | 0.90(0.84,0.96) | <0.001 |
| **Vitamin B12** |  |  | |  |  | |  |  |
| Per 1 unit increment | 1.00(0.99,1.01) | 0.82 | | 1.00(0.99,1.01) | 0.85 | | 1.00(0.99,1.01) | 0.99 |
| **Niacin** |  |  | |  |  | |  |  |
| Per 1 unit increment | 1.00(1.00,1.01) | 0.79 | | 1.00(1.00,1.01) | 0.69 | | 1.00(1.00,1.01) | 0.42 |
| **Total folate** |  |  | |  |  | |  |  |
| Per 1 unit increment | 1.00(1.00,1.00) | <0.0001 | | 1.00(1.00,1.00) | <0.0001 | | 1.00(1.00,1.00) | 0.01 |

Values are weighted OR (95% CI).

Model 1: adjusted for age, sex, and ethnicity.

Model 2: Model 1 + educational level, family income, and marital status.

Model 3: Model 2 + smoking status, alcohol status, physical activity, and HEI-2015.

Abbreviations: CI, Confidence interval; CKM, Cardiovascular-Kidney-Metabolic; HEI-2015, Healthy Eating Index-2015; OR, Odds ratios.

**Table S19** Association between quartiles of B vitamin intakes and advanced CKM syndrome by ordinal logistic regression models.

| **Vitamin B** | Model 1 | |  | Model 2 | |  | Model 3 | |
| --- | --- | --- | --- | --- | --- | --- | --- | --- |
|  | OR (95% CI) | *p* value |  | OR (95% CI) | *p* value |  | OR (95% CI) | *p* value |
| **Vitamin B1** |  |  | |  |  | |  |  |
| Q1 | 1 (reference) |  | | 1 (reference) |  | | 1 (reference) |  |
| Q2 | 0.96(0.79,1.17) | 0.68 | | 0.99(0.81,1.20) | 0.90 | | 0.97(0.79,1.20) | 0.81 |
| Q3 | 0.85(0.71,1.02) | 0.08 | | 0.88(0.73,1.05) | 0.15 | | 0.89(0.74,1.07) | 0.22 |
| Q4 | 0.86(0.73,1.01) | 0.06 | | 0.88(0.75,1.04) | 0.14 | | 0.92(0.77,1.09) | 0.32 |
| *P* for the trend | 0.02 |  | | 0.05 |  | | 0.18 |  |
| **Vitamin B2** |  |  | |  |  | |  |  |
| Q1 | 1 (reference) |  | | 1 (reference) |  | | 1 (reference) |  |
| Q2 | 0.92(0.77,1.10) | 0.35 | | 0.82(0.68,1.00) | 0.05 | | 0.85(0.70,1.03) | 0.10 |
| Q3 | 0.87(0.74,1.03) | 0.10 | | 0.74(0.62,0.88) | <0.001 | | 0.77(0.65,0.92) | 0.01 |
| Q4 | 0.84(0.71,1.00) | 0.05 | | 0.72(0.61,0.85) | <0.001 | | 0.76(0.63,0.90) | 0.003 |
| *P* for the trend | 0.06 |  | | <0.001 |  | | 0.003 |  |
| **Vitamin B6** |  |  | |  |  | |  |  |
| Q1 | 1 (reference) |  | | 1 (reference) |  | | 1 (reference) |  |
| Q2 | 0.79(0.65,0.95) | 0.01 | | 0.73(0.60,0.88) | 0.002 | | 0.72(0.60,0.87) | <0.001 |
| Q3 | 0.89(0.74,1.07) | 0.20 | | 0.81(0.67,0.96) | 0.02 | | 0.79(0.67,0.95) | 0.01 |
| Q4 | 0.78(0.64,0.93) | 0.01 | | 0.78(0.64,0.94) | 0.01 | | 0.77(0.63,0.93) | 0.01 |
| *P* for the trend | 0.04 |  | | 0.05 |  | | 0.04 |  |
| **Vitamin B12** |  |  | |  |  | |  |  |
| Q1 | 1 (reference) |  | | 1 (reference) |  | | 1 (reference) |  |
| Q2 | 1.22(1.03,1.43) | 0.02 | | 1.23(1.04,1.45) | 0.02 | | 1.25(1.05,1.49) | 0.01 |
| Q3 | 1.05(0.89,1.24) | 0.57 | | 1.07(0.90,1.26) | 0.44 | | 1.07(0.90,1.27) | 0.44 |
| Q4 | 1.01(0.83,1.22) | 0.95 | | 1.02(0.84,1.23) | 0.84 | | 1.07(0.88,1.31) | 0.49 |
| *P* for the trend | 0.61 |  | | 0.72 |  | | 0.92 |  |
| **Niacin** |  |  | |  |  | |  |  |
| Q1 | 1 (reference) |  | | 1 (reference) |  | | 1 (reference) |  |
| Q2 | 0.98(0.81,1.19) | 0.84 | | 1.01(0.84,1.23) | 0.89 | | 1.03(0.85,1.26) | 0.74 |
| Q3 | 0.99(0.83,1.17) | 0.88 | | 1.01(0.86,1.20) | 0.87 | | 1.05(0.88,1.24) | 0.60 |
| Q4 | 1.03(0.85,1.24) | 0.78 | | 1.07(0.89,1.29) | 0.48 | | 1.15(0.95,1.39) | 0.15 |
| *P* for the trend | 0.76 |  | | 0.50 |  | | 0.15 |  |
| **Total folate** |  |  | |  |  | |  |  |
| Q1 | 1 (reference) |  | | 1 (reference) |  | | 1 (reference) |  |
| Q2 | 0.85(0.72,1.00) | 0.04 | | 0.88(0.75,1.03) | 0.12 | | 0.91(0.78,1.07) | 0.27 |
| Q3 | 0.68(0.55,0.83) | <0.001 | | 0.71(0.57,0.87) | 0.001 | | 0.74(0.61,0.92) | 0.01 |
| Q4 | 0.62(0.53,0.73) | <0.0001 | | 0.65(0.55,0.77) | <0.0001 | | 0.78(0.65,0.92) | 0.01 |
| *P* for the trend | <0.0001 |  | | <0.0001 |  | | 0.002 |  |

Values are weighted OR (95% CI).

Model 1: adjusted for age, sex, and ethnicity.

Model 2: Model 1 + educational level, family income, and marital status.

Model 3: Model 2 + smoking status, alcohol status, physical activity, and HEI-2015.

Abbreviations: CI, Confidence interval; CKM, Cardiovascular-Kidney-Metabolic; HEI-2015, Healthy Eating Index-2015; OR, Odds ratios.

**Table S20** Effects of mixed B vitamin intakes on advanced CKM syndrome in the sensitivity analyses.

|  | WQS model | |  | QG-comp model | |  |
| --- | --- | --- | --- | --- | --- | --- |
|  | OR (95% CI) | *p* value |  | OR (95% CI) | *p* value |  |
| **Sensitivity analysis 1** | 0.86(0.80,0.93) | 0.0002 | | 0.89(0.83,0.95) | 0.0004 | |
| **Sensitivity analysis 2** | 0.88(0.83,0.94) | 0.0001 | | 0.88(0.83,0.92) | <0.0001 | |
| **Sensitivity analysis 3** | 0.90(0.84,0.97) | 0.005 | | 0.88(0.83,0.94) | <0.0001 | |
| **Sensitivity analysis 4** | 0.89(0.82,0.95) | 0.003 | | 0.87(0.82,0.93) | <0.0001 | |

Abbreviations: CKM, Cardiovascular-Kidney-Metabolic; QG-comp, quantile-based g-computation; WQS, weighted quantile sum.
